# Recent progress in discovery of novel AAK1 inhibitors: from pain therapy to potential anti-viral agents

**DOI:** 10.1080/14756366.2023.2279906

**Published:** 2023-11-13

**Authors:** Ying-Hui Yuan, Nian-Dong Mao, Ji-Long Duan, Hang Zhang, Carmen Garrido, Frédéric Lirussi, Yuan Gao, Tian Xie, Xiang-Yang Ye

**Affiliations:** a School of Pharmacy, Hangzhou Normal University, Hangzhou, Zhejiang, China; b Key Laboratory of Elemene Class Anti-Cancer Chinese Medicines; Engineering Laboratory of Development and Application of Traditional Chinese Medicines; Collaborative Innovation Center of Traditional Chinese Medicines of Zhejiang Province, Hangzhou Normal University, Hangzhou, Zhejiang, China; c School of Basic Medical Science, Hangzhou Normal University, Hangzhou, China; d INSERM UMR 1231, Labex LipSTIC, University of Bourgogne, Dijon, France; e University of Franche-Comté & University Hospital of Besançon, Besancon, France; f Cancer Center George François Leclerc, Dijon, France; g University of Bourgogne Franche-Comté, Besançon, France; h Institute of Chinese Materia Medica, Shanghai University of Traditional Chinese Medicine, Shanghai, China

**Keywords:** Adaptor associated kinase 1 (AAK1), inhibitor, medicinal chemistry, therapeutic application

## Abstract

Adaptor associated kinase 1 (AAK1), a member of the Ark1/Prk1 family of Ser/Thr kinases, is a specific key kinase regulating Thr156 phosphorylation at the μ2 subunit of the adapter complex-2 (AP-2) protein. Due to their important biological functions, AAK1 systems have been validated in clinics for neuropathic pain therapy, and are being explored as potential therapeutic targets for diseases caused by various viruses such as Hepatitis C (HCV), Dengue, Ebola, and COVID-19 viruses and for amyotrophic lateral sclerosis (ALS). Centreing on the advances of drug discovery programs in this field up to 2023, AAK1 inhibitors are discussed from the aspects of the structure-based rational molecular design, pharmacology, toxicology and synthetic routes for the compounds of interest in this review. The aim is to provide the medicinal chemistry community with up-to-date information and to accelerate the drug discovery programs in the field of AAK1 small molecule inhibitors.

## Introduction

Diseases of the central nervous system, such as neuropathic pain, Alzheimer’s disease (AD), and Parkinson’s disease (PD), seriously affect human health and social development, and have become serious public health problems around the world. Among them, Alzheimer’s disease and Parkinson’s disease are characterised by progressive degeneration of specific neurons in the brain, which currently affect many people in China^1^. However, at present, the treatment of central nervous system diseases is still a major medical problem in the world. Adaptor Associated Kinase 1 (AAK1) plays an important role in the treatment of nervous system diseases. AAK1, also known as AP-2-associated protein kinase 1, belongs to the Numb-associated kinase (NAK) family^2^^,^^3^. It is a highly conserved Ser/Thr protein kinase^4^^,^^5^. AAK1 was first identified in α-adaptin protein-interacting partners associated with phosphorylating the μ2 subunit of AP-2 at Thr156 and playing a regulatory role in the process of CME^6^^,^^7^. The reason why AAK1 can play an important role in nervous system diseases is that AAK1 participates in many nervous system-related signal pathways, such as NDR and CDK signal pathways. Therefore, AAK1 is associated with many human diseases, such as neuropathic pain, AD^8^, PD^9^, amyotrophic lateral sclerosis (ALS)^10^ and so on. At present, several AAK1 inhibitors have entered the clinical stage, such as BMS-986176/LX-9211 for diabetic peripheral neuropathic pain (NCT04455633) and post-herpetic neuralgia (NCT04662281)^11^.

It is worth noting that with the outbreak of COVID-19 in recent years, AAK1i has shown significant clinical efficacy in the treatment of COVID-19^12–14^. Increasing evidence shows that drugs such as Baricitinib, Erlotinib, Sunitinib, and Fedratinib on the market have the ability to target AAK1 activity and thus play a role in blocking virus endocytosis^15^. The main mechanism is that AAK1 can phosphorylate AP2M1, which is very important for the virus to induce clathrin-mediated endocytic pathway into cells. And in order to reduce virus invasion, the activity of the AAKl is inhibited and phosphorylated AP2M1 is down-regulated in AAKli. Long before the treatment of coronavirus, Neveu et al. proposed that AAK1 inhibitors have the effect of hepatitis C virus (HCV) in 2012^16^. Subsequently, it was clearly stated in the US patent filed by Einav et al. that AAK1 inhibitors can destroy the assembly of HCV and the replication of HIV-1^17^. However, the anti-viral effect of AAK1 seems to have been ignored by scientists, and its related research has not been reported in the literature. Until the outbreak of COVID-19 in 2019, researchers actively explored various mechanisms of antiviral treatment, and paid more attention to the anti-viral effect of AAK1 inhibitors.

Over the past several decades, research involving AAK1 has proliferated, as evidenced by the large numbers of scientific publications on the topic. Literature using the SciFinder® search engine and PubMed website with “AAK1” or “adaptor associated kinase 1” as the key word provide all references for further analysis. These references were further analysed by publication year. The result reveals that the publication (papers and patents) number increased from 19 in 2005 to 429 in 2023. Then the search was conducted again using “inhibitor” to exclude those references focuses on pure biology without involving any AAK1 inhibitors. The references obtained above were then further categorised using the therapeutic area as criteria. Therapeutic applications of AAK1 inhibitors that are being identified or validated mainly focus on pain and neurological diseases^18^, and anti-viral compounds^19^. The outbreak of the COVID-19 epidemic in recent years significantly increased the awareness of AAK1 protein and contributed to the hike in publication number. Recent advances in treating sequelae of COVID-19 infection suggest AAK1 could become one of the potential therapeutic targets to treat COVID-19^12–14^. drug discovery/drug discovery fields, a number of articles have been published in different major journals. However, there are few articles about AAK1 inhibitors^20^^,^^21^. In terms of drug discovery targeting AAK1 protein with small molecule inhibitors, relevant work has been carried out in this research group for several years. In addition, a literature review of AAK1 inhibitors was initiated last summer to formulate a review of the field with a focus on the medicinal chemistry aspect. This updated summary of accomplishments in the field could ultimately be helpful to the drug discovery community And a detailed analysis of AAK1 inhibitors from biochemical concepts to design strategies has been conducted, with the aim of formulating a field review focusing on medicinal chemistry. It is worth believing that the latest summary of achievements in this field may eventually help the drug discovery community. And the hope is that it will serve as a quick reference guide for the scientific community and inspire new ideas to accelerate drug discovery in this field.

## Structural and biological function of AAK1

### Structure of AAK1

**Figure 1.**
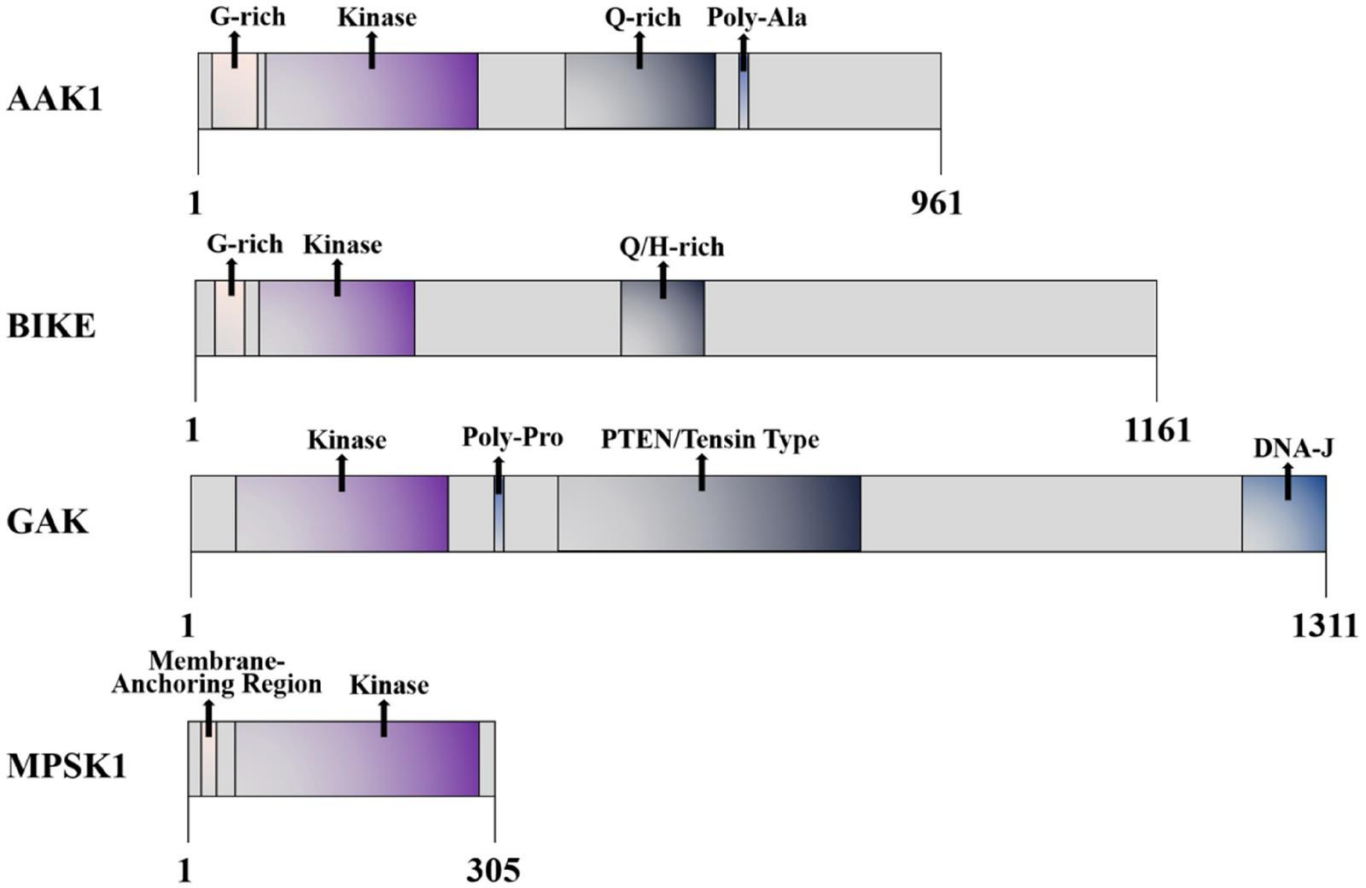
Schematic diagram of the AAK1 kinase domain and domain organisation comparison of the human NAK family.

AAK1 is a Ser/Thr protein kinase that belongs to the numb-associated kinase (NAK) family. This family also includes serine/threonine kinase 16/myristoylated and palmitoylated serine/threonine kinase 1 (STK16/MPSK1), Cyclin G associated kinase (GAK), and bone morphogenic protein BMP-2 inducible kinase (BIKE)^22^^,^^23^. And AAK1 is composed of 868 amino acids, with a highly conserved kinase domain (about 267 amino acids) at the N-terminal, α-aptamer interaction domain (AID) at the C-terminal, and a glutamine (28%) -proline (18%) -alanine (13%) QPA-rich region (residues 312–630) in the middle region (Figure 1)^3^^,^^7^. Moreover, a long form of AAK1 (AAK1L) containing an extended C-terminal has been identified^24^. This kind of AAK1 encodes an additional grid protein binding domain (CBD2), which consists of several low-affinity interaction motifs. However, although AAK1L CBD2 can directly bind to grid protein, its overexpression impairs the endocytosis of transferrin. Moreover, the fact that AAK1 plays a role in multiple steps of the endosome pathway has also been confirmed.

In the NAK family, kinase domains are all close to the amino terminal and highly similar, but they have their own characteristics in other regional domains^4^. This fact highlights the substrate specificity and the different physiological functions of each family member. For example, NAK is related to a wide range of cell functions. In addition to the previously-mentioned functions of binding to clathrin and phosphorylating the AP-2 μ2 subunit, NAK promotes Notch activation that regulates signal transduction between Notch cells. AAK1 is also a substrate for NDR1/2 phosphorylation that has been shown to control dendrite morphogenesis in developing mammalian neurons^25^. However, it has been reported that the AAK1 complex can be knockdown by RNAi approaches. In this study, it is demonstrated that AAK1 silencing affects the expression of epithelial-mesenchymal plasticity (EMP) markers and greatly improves the sensitivity of cancer cells to targeted therapy^26^. This underscores the importance that AAK1 may also have in the treatments of cancers and other diseases.

BIKE is closely related to AAKl in structure, which is related to Numb. It plays a role in osteoblast differentiation and has recently been identified as a clathrin-coated vesicle-associated protein^27^^,^^28^. BIKE also negatively regulates polyploidy and megakaryocyte differentiation in acute megakaryocytic leukaemia by interacting with cyclin-dependent kinase 2 (CDK2) and promoting mitosis^29^. GAK is a known association partner of cyclin G and CDK5, some of its known functions are shared with AAKl. GAK is necessary for reticulin transport, and mediates the binding with plasma membrane and *trans*-Golgi network. GAK is necessary for maintaining centrosome maturation and progress through mitosis^30^. GAK has been reported to be involved in chronic kidney disease (CDK) and has been discussed as a potential drug target for the treatment of viral infections due to its involvement in the entry and replication of multiple viruses^31^. MPSK1 is the most distantly related member of the AAK1 family, and its physiological function is still poorly understood. It is known to be a Golgi-related kinase, which plays an uncertain role in regulating secretion in the constitutive secretion pathway of the *trans*-Golgi network^32^. MPSK1 is also related to mammary gland development in mice^33^.

**Figure 2.**
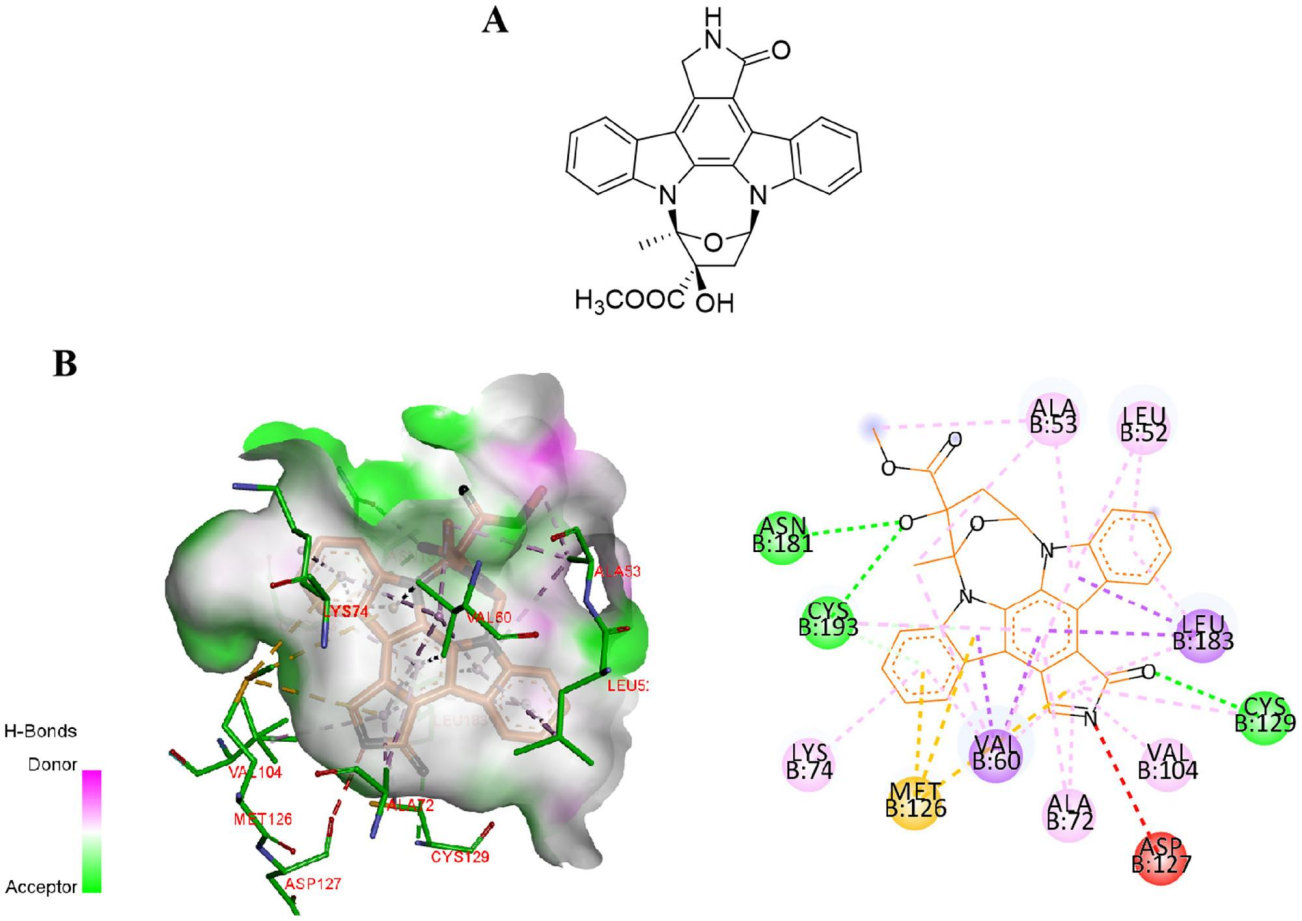
(A) The chemical structure of K252a. (B) Interaction of K252a with AAK1 (protein data bank (PDB) ID: 4WSQ). Inhibitors are shown in stick representation with yellow carbon atoms. Key interacting residues are shown and labelled.

Kuai et al. found that K252a, a natural compound containing indolocarbazole functionalities (tryptophan sugar compounds), enhances the signal transduction and neurogenesis of Neuregulin-1 (Nrg1)/ErbB4-dependent neurotrophic factor by integrating chemical genomics and proteomics. However, the deletion of AAK1 as the related target of K252a affects the transport and expression level of ErbB4, which provides evidence for AAK1 in Nrg1-mediated neurotrophic factor signal transduction^34^. In 2016, Sorrell et al. successfully resolved the eutectic structure of the AAK1/K252a complex^3^ (Figure 2). Subsequently this structure has provided a strong rationale for the design and optimisation of AAK1 inhibitors. Small molecule inhibitors K252a with a near-atomic resolution of 1.95 Å. They crystallised in space group *P*2_1_2_1_2_1_, wherein each asymmetric unit (chain A and B) has two AAK1 kinase domains (Figure 2). In chain A, the C-terminal forms a part of the helix, extending outward from the kinase domain into the solvent channel of the crystal complex. Through gel filtration and crystallography, they showed that AAK1 chiefly exists in monomeric form rather than dimeric, with a typical bi-lobal catalytic domain structure. The details of AAK1’s crystal structure include the localisation of the activated C-terminal helix (ASCH), and the position of zinc atoms present at high concentration in the crystallisation solution. All are shown in relation to the symmetry-related molecule (Sym 1).

In AAK1, hydrogen bonding residues Gln203 interact with the N-terminal of ASCH and the αE/αF loop on Ile239. Residues 204–208 in AAK1 (and equivalent residues in BIKE) form a short turn through intramolecular interactions to provide greater conformational change. In addition, the kinase Ala-Pro-Glu (APE) motif (Glu229 in AAK1) forms a salt bridge that stabilises the C-terminal loop. The residues Tyr225 and Arg226 before the APE sequence in AAK1 help to lock the activated fragment in a certain position. AAK1 is a constitutively active kinase because Met94 of α-C helix, Tyr106 of β4, and His174 and Phe195 of HRD and DFG motifs constitute the regulatory spine (R spine) that keep it aligned. In addition, there is a flexible phosphate-binding loop at the ATP site in AAK1, called the “P ring”, which is located in Ala53-Ala59 of AAK1. Adding a glycine at the X_2_ position in the sequence G-X_1_-G-X_2_-φ-G allows this sequence to become a di-glycine motif that changes the conformation of the loop to adapt to various inhibitor scaffolds^3^.

### Biological function of AAK1

**Figure 3.**
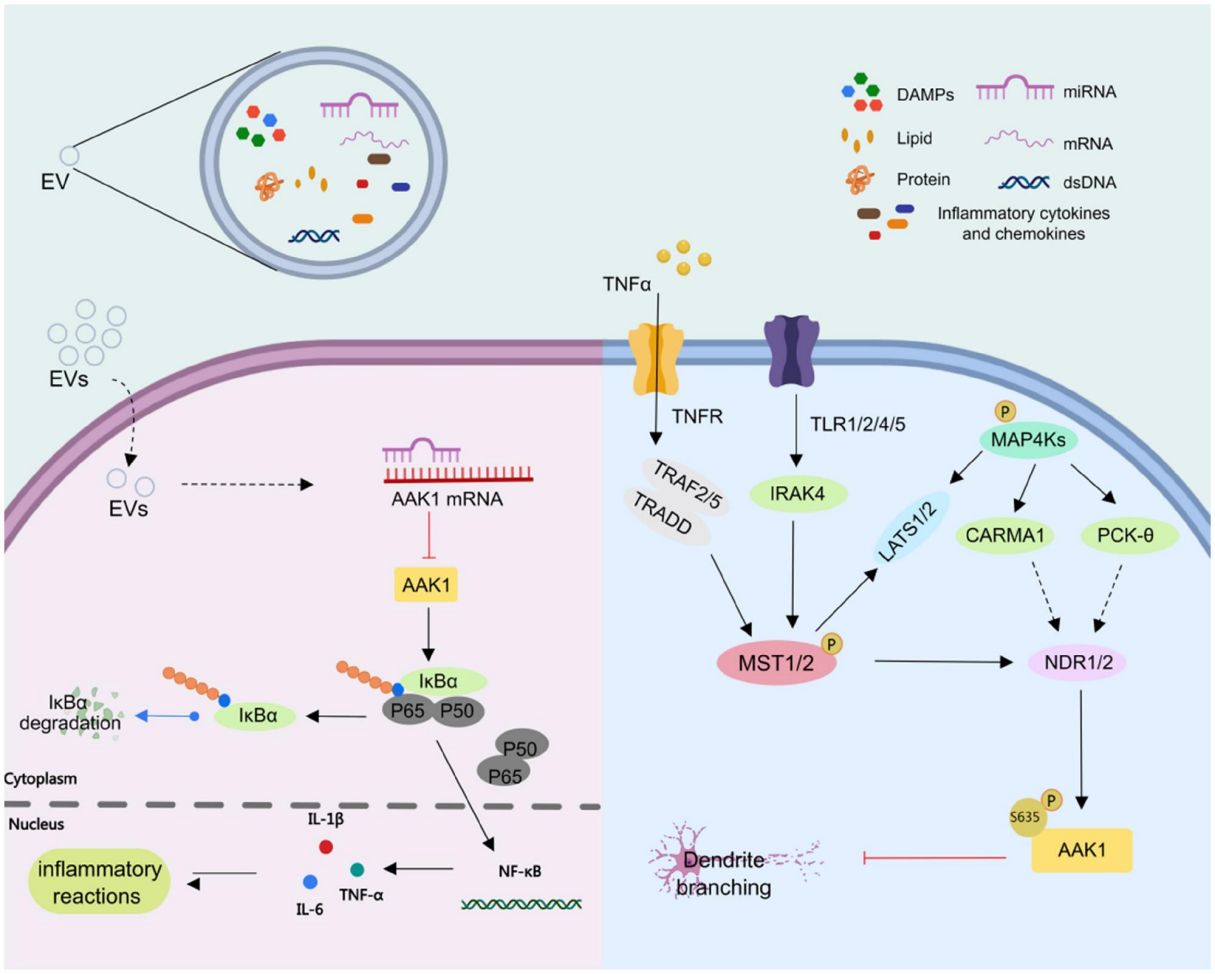
AAK1 medicates NF-κB and NDR1/2 signalling pathway. Left, AAK1 accelerates the degradation of IKBα, which leads to the activation of trans-acting factor p50/p65 and the transcription of pro-inflammatory cytokines, such as IL-6, TNF-αand IL-1β. Right, the Ser635 phosphorylation of AAK1 is regulated by phosphorylated NDR1/2, which was controlled by the upstream kinases MST1/2 and MAP4Ks. Phosphorylated AAK1 inhibits the growth of dendritic branches.

AAK1 is implicated in multiple cellular pathways. For example, AAK1 participates in the NF-κB signalling pathway. AAK1 mediates IKBα to combine with inhibitors and forms a trimer p50-p65-IKBα complex, which becomes phosphorylated (Figure 3). Under catalysis by Skp1-cullin 1-F-box (SCF) E3 ligase complex (SCF-E3 ubiquitinase), IKBα becomes ubiquitinated and degraded by a protease. The activated NF-κB is transferred to the endosome where it combines with its related DNA motifs to induce transcription of the target gene. This leads to degradation of target cellular proteins, including signal transducers, cell cycle regulators, and transcription factors. Recruitment of the target gene produces widespread immune and inflammatory reactions (Figure 3). Lian et al. explained that miR-671-5P transmitted by MenSCs through extracellular vesicles (EVs) directly degrades AAK1. This results in inhibition of the activation of NF-κB and the signalling pathway mediated by AAK1^35^. In addition, Abhinand et al. reported that AAK1-AP2M1 regulates vascular endothelial growth factor (VEGFR) by endocytosis^36^. The combination of VEGFA-165 and VEGFR2 activates other pathways such as RAS-MAPK, PI3K-AKT-mTOR, PLC-PKC, JAK-STAT, NF-κB, p38MAPK-FAK and WAK. The interaction between these pathways contributes to the proliferation, migration and survival of endothelial cells and the formation of tubes involved in angiogenesis^37^.

In addition, AAK1, as the substrate of NDR1/2 kinase, a key NDR family kinase in dendrite regulation, plays an important role in dendrite branching and dendritic spine growth^25^. The phosphorylation of NDR1/2 is regulated by an upstream gene involving phosphorylated MST1/2 and MAP4Ks (Figure 3)^21^^,^^38^. NDR1/2 is intimately involved in the process of polarised dendrite growth in an evolutionary conserved pathway from yeast to mammals. It is known to control growth and morphology in worm, fly, and mammal. Moreover, it is clear that NDR1/2 phosphorylation of AAK1 at Ser635, inhibits the growth of dendrite branching and arborisation (for a recent review see Ref. 21). AAK1 is not only implicated in pathways linked to neurodevelopment but also is involved in the regulation of the Notch pathway.

**Figure 4.**
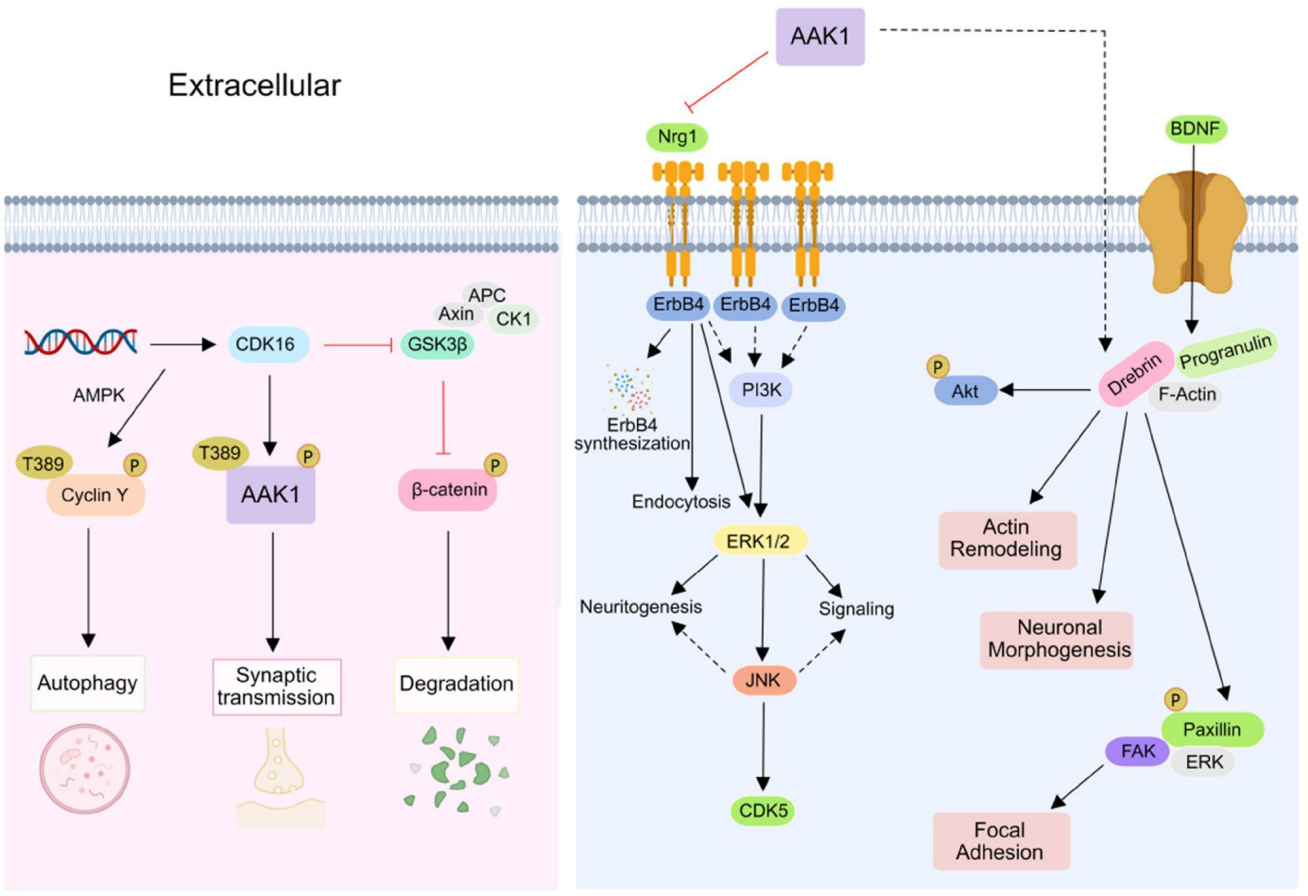
AAK1 participates in CDK16 and Nrg1 signalling pathway. Left, the phosphorylation of AAK1 Thr389 was controlled by CDK16, which was involved in the regulation of neuronal synaptic transmission. Right, AAK1 inhibits the Nrg1/Erbb4-dependent neurotrophic factor signal transduction. Inhibition of AAK1 results in the sustained activation of downstream signalling by ErbB4, leading to enhanced Nrg1-mediated neurogenesis.

The Notch signalling pathway widely exists in vertebrates and invertebrates and is highly conserved in evolution. It regulates the differentiation and development of cells, tissues, and organs through the interaction between adjacent cells, and is related to many human diseases^39^. It is reported that γ-Secretase processing of Notch is essential in mono-ubiquitination and clathrin mediated endocytosis (CME). In addition, Eps15 contains many isoforms, such as Esp15b and its related protein Eps15R, which have been confirmed to participate in reticulin-mediated endocytosis, and the protein containing UIM is known to interact with the ubiquitination receptor, and might thus be involved in Notch endocytosis complex. Gupta-Rossi et al. confirmed that AAK1 acts as an adaptor for a Notch interaction with components of the clathrin-mediated pathway Eps15b^40^. Transfected AAK1 increases the localisation of activated Notch cell types to produce Rab5-positive endocytic vesicles. On the other hand, AAK1 depletion or overexpression of Numb, an inhibitor of the pathway, interferes with this vesicle localisation and distribution. These results suggest that after ligand-induced activation of Notch, the membrane-tethered form is directed to different endocytic pathways leading to distinct fates. AAK1 also activates the WNT pathway and promotes the synthesis and transmission of the regulating factor Rab5 in early and late stage endosomes. In addition, AAK1 is activated by Numb. In turn, Notch receptors are inhibited by AAK1. This indicates that there is a dynamic balance between AAK1 and the Notch receptor^21^ (Figure 4). WNT promotes the endocytosis of the WNT receptor (LRP6) in a reticulin fibre meshwork. It activates AAK1 and phosphorylates the Thr156 site of AP2M1. However, the abundance of LRP6, which is also an inhibitor of WNT, thus forms a negative feedback loop driven by AAK1^38^. In addition, AAK1i stabilises β-catenin and activates the β-catenin-dependent WNT signal pathway that accelerates the transcription of the target gene^41^^,^^42^ (Figure 4).

**Figure 5.**
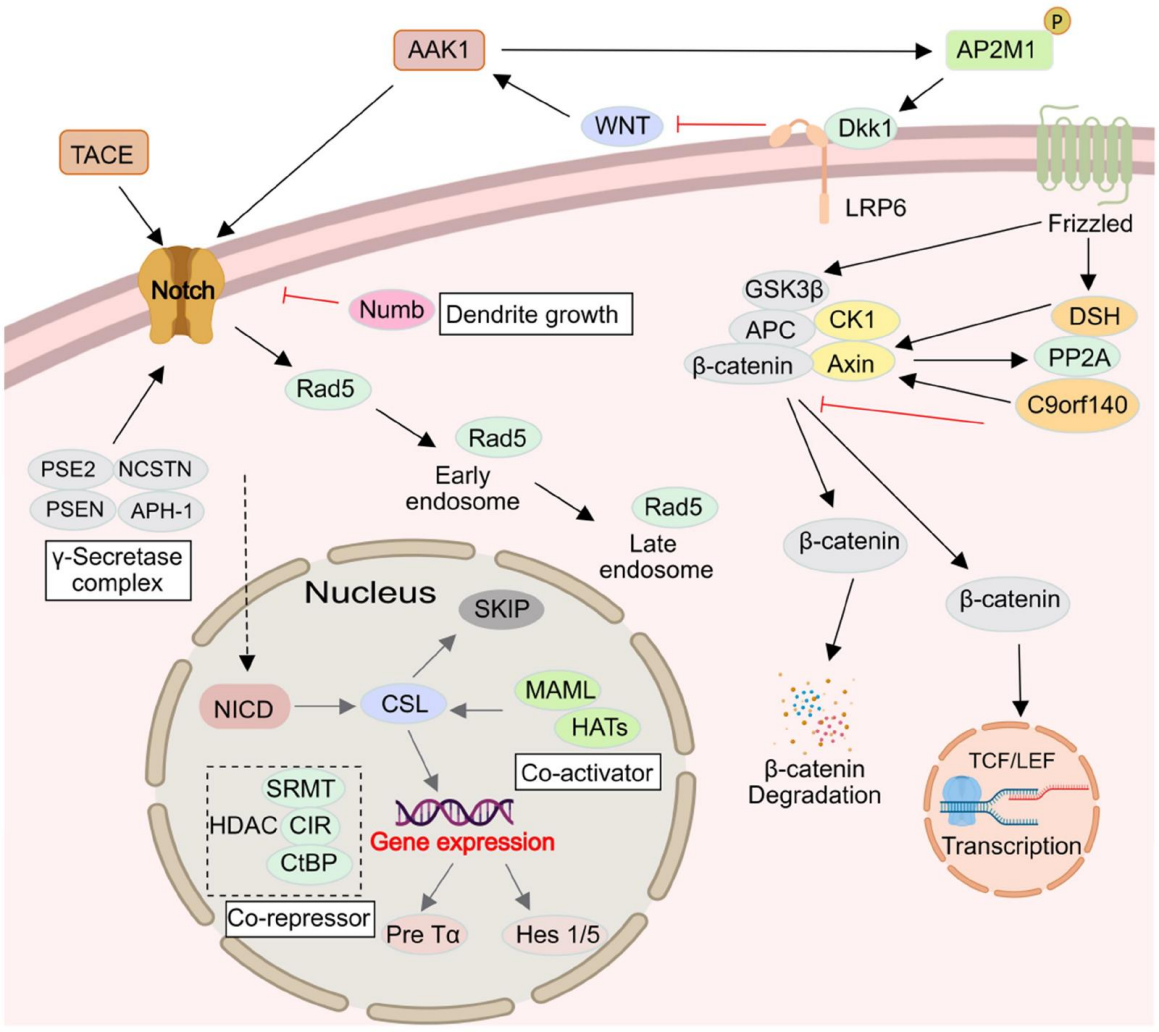
AAK1 is involved in Notch and WNT signalling pathway. Firstly, AAK1 mediates the interaction of Notch and Eps15b, which accelerates the Notch pathway. Secondly, AAK1 negatively regulates WNT signalling by promoting CME of LRP6. Meanwhile, WNT activates AAK1-activated AP2M1 phosphorylation to promote endocytosis.

AAK1 also plays a role in the cyclic AMP dependent CDK16 signal pathway that regulates the phosphorylation of PRC1 in lung cancer cells. As a substrate of CDK16, AAK1 also plays a potential role in neuron and brain diseases. The abundance of MiR-125b-5p is known to affect the activity of CDK16, thus indirectly inhibiting cell degradation and promoting autophagy. CDK16 promotes synaptic transmission by phosphorylating the Thr389 site of AAK1 (Figure 5)^43^. AAK1 also inhibits the Nrg1/ErbB4-dependent neurotrophic factor signal transduction. By activating ERk1/2, the archetypical kinase enhances neurogenesis and signal transduction. At the same time, other ErbB receptors may also be susceptible to inhibition by AAK1^34^. AAK1 regulates brain-derived neurotrophic factor (BDNF) that enhances the interaction between paxillin and drebrin may promote neuronal morphogenesis and actin remodelling^44^.

## AAK1 and human diseases

**Figure 6.**
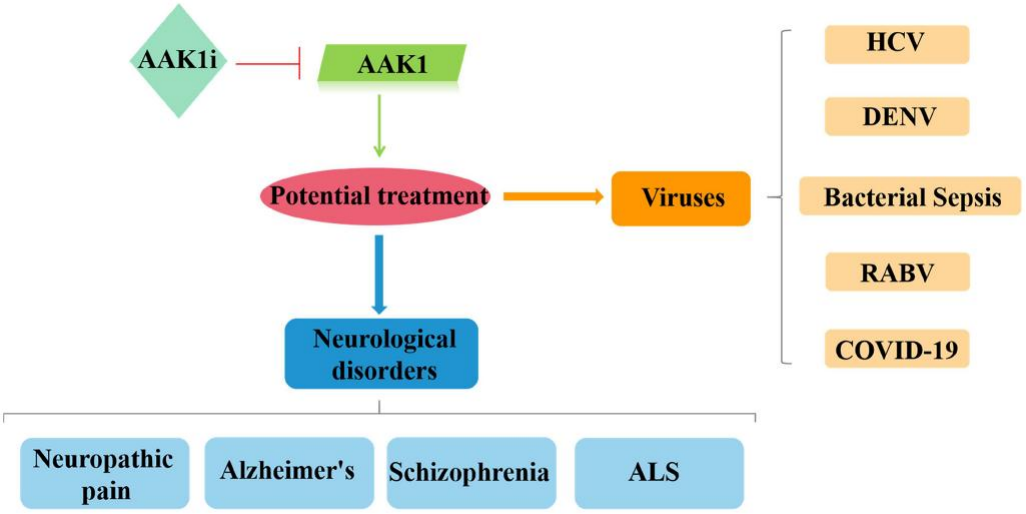
Relevance of AAK1 protein and therapies for human diseases. When AAK1 kinase is inhibited, it has a potential therapeutic effect, which is associated with neurological disorders and antiviral activity.

Due to the important biological functions of AAK1, studies of AAK1 as a potential therapeutic target have been vigorous and fruitful over the past few decades. Indeed, intervention in AAK1 phosphorylation has been shown useful in clinical trials for the treatment of neurological diseases such as neurological pain, AD, Schizophrenia, and ALS. Intervention in AAK1 catalysis has also been explored for potential therapies in the treatment of viral diseases including HCV, dengue virus and ebola virus (DEVN), bacterial sepsis, rabies virus (RABV), and COVID-19 (Figure 6).

### AAK1 and neurological diseases

Because endocytosis is closely related to many pathophysiological functions of the nervous system, and AAK1 closely regulates the steps endocytosis, we hypothesise that AAK1 may affect cellular function by participating in the pathological development of some nervous system diseases. Scientists have provided many studies to examine this point.

Neuropathic pain, which is associated with poor quality of life, has huge unmet clinical needs. However, due to a variety of side effects, limited scope of treatment and unsatisfactory treatment, only a few new painkillers have entered the market in the past decade. Therefore, new drugs with higher efficacy and fewer adverse reactions are needed to treat neuropathic pain. In this regard, AAK1 is a new feasible target for the treatment of neuropathic pain, and it has been confirmed for the first time in mouse knockout phenotype. Kostich et al. reported AAK1 as a novel target for the treatment of neuropathic pain^45^. And it found that AAK1 knockout mice showed a significantly reduced response to persistent pain in the formalin test and had no mechanical ectopic pain within 3 weeks after spinal nerve ligation. In addition, a potent small molecule inhibitor of AAK1 (LP-935509, brain to plasma (B/P) ratio >2) was also identified. It could also reduce chronic contractile injury (CCI) in a rat model of diabetic peripheral neuropathy and reduction of pain responses in a rat streptozotocin model^45^. Moreover, Luo et al. found that BMS986176/LX9211 possesses good efficacy for pain remediation in two rodent neuropathic pain models. With the completion of phase I clinical studies, this effort in humans will continue. Louis et al. developed a potent and selective AAK1 radio-ligand, [^3^H]BMT-046091, and observed the distribution of AAK1 binding sites in the brain and spinal cord of rodents and cynomolgus monkeys^46^. The results showed that AAK1 is widely expressed in the brain and spinal cord, with the highest expression level in the forebrain. In addition, orally bioavailable LP-935509 could be found to occupy the AAK1 binding site in a dose-dependent manner. And the target occupancy of LP-935509 was correlated with its anti-diabetic pain effect^46^. In 2022, Chen et al. demonstrated that miRNA-384-3p attenuated sevoflurane-induced neuronal apoptosis and memory impairment. By proposing that this compound inhibits the expression of AAK1, the effect of mitigating sevoflurane-induced nerve damage in paediatric clinical surgery was demonstrated^47^.

Apart from intensifying painful neuronal responses, AAK1 has been shown to promote amyloid β (Aβ)-induced damage in AD through the intervention of the AP2M1 adapter. AAK1 co-locates with endosomal and presynaptic protein markers, but it is wrongly identified as an aggregate containing mutant SOD1 and neurofilament protein in ALS, which complex inhibit the activity of AAK1. In 2018, Fu et al. used Aβ1-42 to establish a mouse AD model and used the Morris water maze test to verify that the periodic changes in AAK1, AP-2, and Rab-5 (early intranuclear marker) expression were closely related to the decline of cognitive ability associated with AD^8^.

### AAK1 and viral diseases

**Figure 7.**
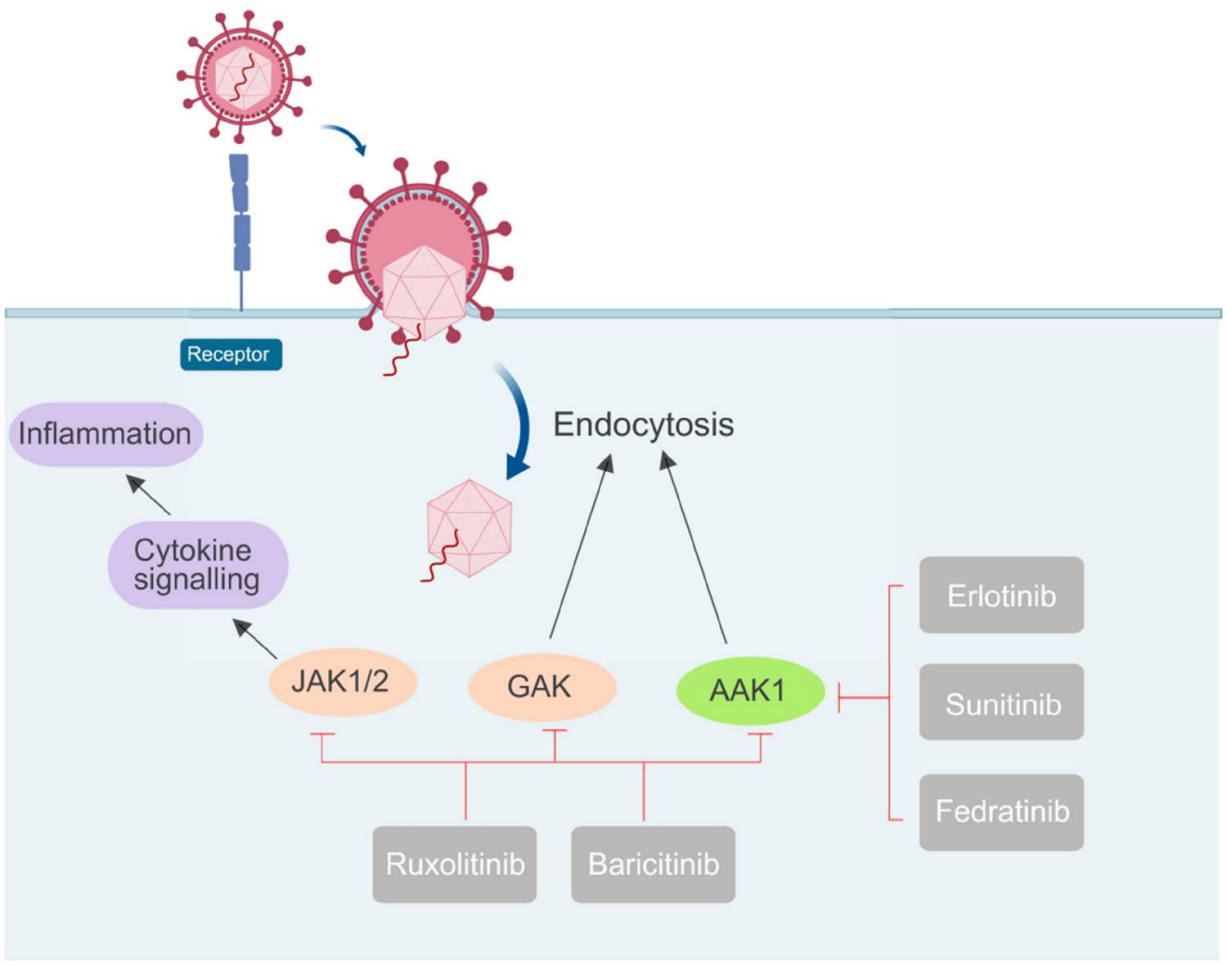
Different drugs currently on the market that act on JAK1/2, GAK, and AAK1 kinases to prevent virus invasion, mainly to inhibit the endocytosis of the virus.

Viruses, capable of infecting all organisms from bacteria, plants, animals to humans, are a major source of infectious disease. Viral infections have caused huge burdens and inflicted damage to human health and economic activities through the ages. AAK1 was generally considered as a feasible biological target for the treatment of neuropathic pain, but recently, scientists have found that AAK1 may also be a potential anti-viral target. In the review by Huang, the role of the Numb-associated kinases family in anti-virus therapy was also mentioned, including AAK1 in April 2023^48^. Recently, scientists have found that inhibiting AAK1 can potentially regulate endocytosis, thereby inhibiting virus invasion into cells (Figure 7).

Sunitinib and Erlotinib are new multi-targeted tyrosine kinase inhibitors, which were first approved for the treatment of cancer. The dissociation constants (K*_d_*) of Sunitinib and Erlotinib for binding AAK1 to GAK are 11 nmol L^−1^ and 3.1 nmol L^−1^, respectively^49^. In 2015, further studies confirmed that AAK1 and GAK were the key regulators of HCV entry into cells, and the anti-viral effects of Sunitinib and Erlotinib might contribute by mechanisms involving the inhibition of AAK1 and GAK^50^. In 2017, Einav’s group reported that the roles of AP-1 and AP-2 complexes, as well as AAK1 and GAK, in the entry, assembly, and release of Flaviviridae family members are significant. It is further confirmed that these host factors represented a broad-spectrum of anti-inflammatory agents that were potential targets for anti-viral therapy. In addition, compared to direct-acting anti-viral drugs, administering the Sunitinib/Erlotinib combination could potentially provide greater resistance against DENV. This combination limited DENV infection *in vitro* and reduced the related viremia, morbidity, and mortality in a mouse model. It was also found that the combination of tyrosine kinase inhibitors blocked the entry and late stages of the life cycle of four DENV serotypes and multiple viruses^51^.

**Figure 8.**
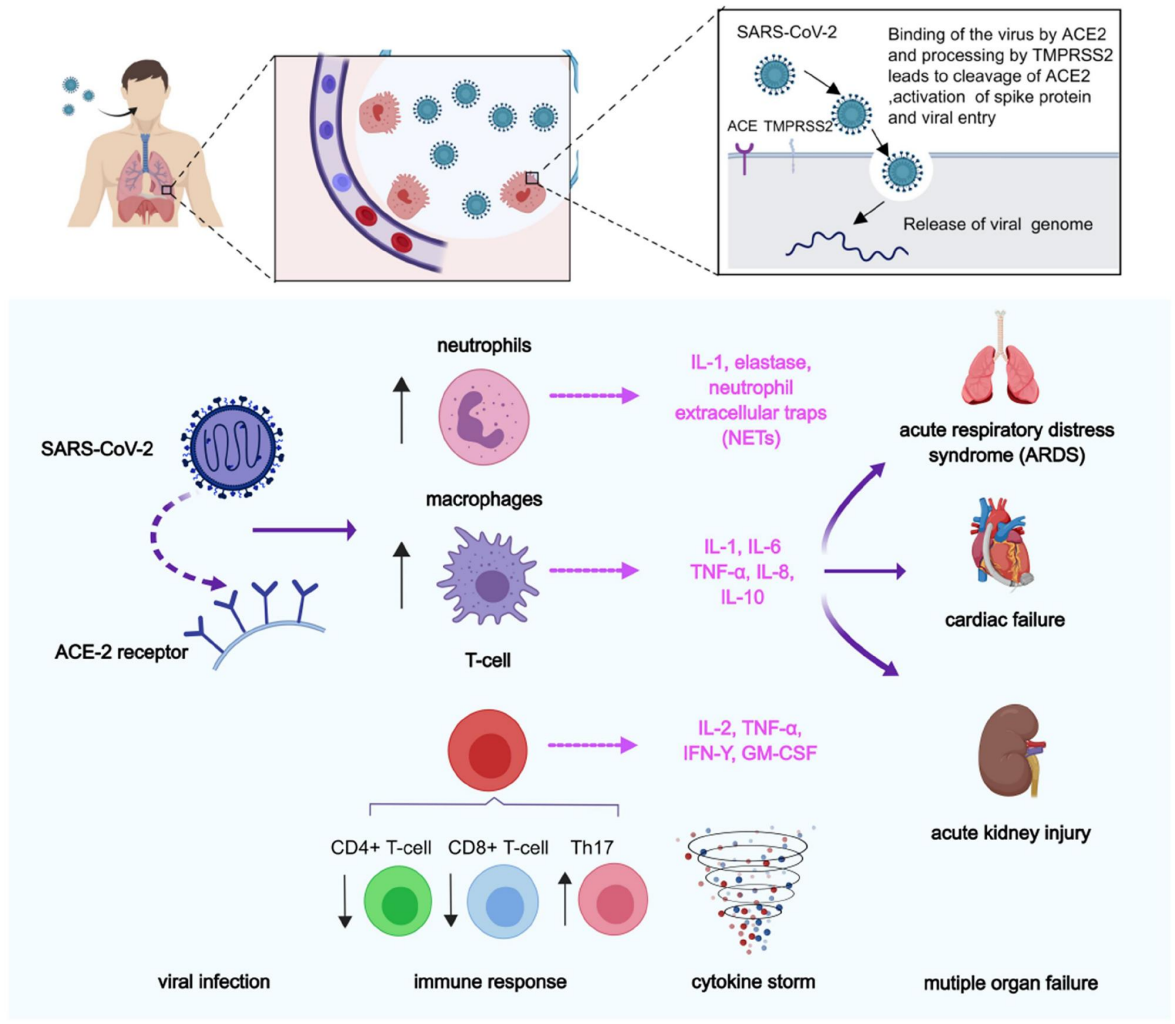
Pathological process after SARS-CoV-2 infection. First, SARS-CoV entered into lung epithelial cells, by binding of the virus to the ACE-2 receptor via spike proteins expressed on the surface of the virus. After the viruses proliferated in large numbers, the viruses can be recognised by neutrophils, macrophages, or T-cells. The inflammatory responses were triggered and the danger signalling molecules such as certain cytokines (e.g. IL-1, IL-8, etc.) were producted. In addition, immune response were elicited by reducing the number of CD4+ T-cells and CD8+ T-cells and increasing the number of Th17 cells.

Angiotensin-converting enzyme 2 (ACE2) is the primary receptor for the COVID-19. It was recently reported that inhibition of the ACE2 receptor provides a new therapeutic direction in fighting COVID-19 and reducing its mortality^52^. In addition, inhibition of the transmembrane protease, serine 2 (TMPRSS2) and cathepsin L (CTSL), of the endocytosis regulator AAK1, and of the pro-inflammatory cytokines- IL-6, IL-1β, TNF-α, and IFN-γ, all significantly reduce mortality and morbidity associated with COVID-19 infection (Figure 8). Also, Akbarzadeh-Khiavi et al. summarised two possible mechanisms of cell entry of SARS-CoV-2^53^. The first is that the virus binds to the ACE-2 receptor outside the plasma membrane, and the endocytosis process occurs by assembling clathrin and coat proteins on the inner side of the plasma membrane in the form of a vesicle. The second mechanism presupposes that SARS-CoV-2 directly uses clathrin-dependent endocytosis to enter host cells. Following viral entry, viral RNA is released into the cytoplasmic space to initiate the production of structural and non-structural viral proteins. Finally, virions of coronaviruses are formed by budding into the endoplasmic reticulum-Golgi intermediate compartment and released as new virions. AAK1 may affect the assembly and entry of the SARSCoV-2 virus through the operation of one of these two possible mechanisms. Later studies by Ou et al. showed that endocytosis is the preferred pathway for SARS-CoV-2 to enter host cells^54^. In addition, The Kar et al. paper also suggests a new method for the treatment of COVID-19. Kar et al. analysed the inhibitory potential of 605 plant compounds, selected from Indian medicinal plants with reported antiviral and anti-inflammatory activities, against the receptor-binding domain of spike proteins of the SARS-CoV-2 wild-type and the variants of concern, including variants B.1.1.7 (Alpha), B.1.351 (Beta), P0.1 (Gamma), B.1.617.2 (Delta), and B.1.1.529 (Omicron)^55^. On the other hand, they found taraxerol antagonises the human ACE2, CTSL, and TNF-α. β-amyrin antagonises the human TMPRSS2. Cynaroside antagonises the human AAK1 and IL-1β. Friedelin antagonises the human IL-6 and IFN-γ.

Janus kinase (JAK) is a family of intracellular, non-receptor tyrosine kinases that transduce cytokine-mediated signals via the JAK-STAT pathway. Baricitinib, an effective and selective JAK inhibitor, is currently used to treat rheumatoid arthritis (RA). However, recently Baricitinib has been considered as a potential therapeutic drug for COVID-19 because of its anti-inflammatory and anti-viral activities, which may prevent the abnormal production of pro-inflammatory cytokines commonly observed in COVID-19 patients. In addition, Baricitinib also inhibits the virus from entering the target cells by binding AP2-related protein kinase 1 (AAK1). In 2020, Lo et al. reported for the first time the favourable clinical course and outcome of an RA patient who had been treated with Baricitinib for more than one year and who developed COVID-19. The patient received supplemental oxygenation, in addition to lopinavir/ritonavir, hydroxychloroquine, while continuing to use Baricitinib. After recovery, the treatment was judged to be successful. She is currently apyretic with an O_2_ saturation 95% in ambient air. While his family received the same treatment except for Baricitinib, his condition deteriorated rapidly, and he died of respiratory failure a few days later^56^. Although the clinical data here are only preliminary, they provide strong support for further study of the use of Baricitinib in the treatment of COVID-19. When the pandemic ended, a large number of investigators employed artificial intelligence (AI) algorithms to independently predict and evaluate *in vitro* pharmacology and *in vivo* pharmacokinetics of Baricitinib in related leukocyte subsets. Through analysis of reported biochemical and cellular results, this series of numerous case studies confirmed the anti-cytokine signal transduction behaviour and reported the antiviral effect of Baricitinib^52^. This treatment may be related to the clinical and radiological recovery, the rapid decline of SARS-CoV-2 viral load, drop in inflammatory markers and IL-6 levels^51^. In a series of clinical I/II randomised controlled studies in 2021, González et al. confirmed that Baricitinib could prevent respiratory insufficiency in tumour and hematological disease patients with COVID-19^57^. Also, the drug Ruxolitinib has displayed clinical effectiveness in COVID-19 patients^58^. The above results prove that effective anti-viral drugs can be found from other existing drugs. Because the proteins in the host have complex functions and various mechanisms of action, they cannot be used for treatment rashly. Moreover, considering the toxicity problem, the *in vitro* experiment can not be applied to the *in vivo* experiment.

## AAK1 inhibitors for the treatment of neuropathic pain

AAK1 inhibitors were first used to treat neuropathic pain, and several inhibitors have entered clinical research. The interaction mode between inhibitors and AAK1 kinase can be realised by X-ray crystal diffraction, which will be beneficial to further structural optimisation. Herein the latest research progresses on different categories and different chemical scaffolds of small molecular AAK1 inhibitors were summarised with the hope to provide a reference for the treatment of pain and to accelerate the drug discovery in this field.

### Imidazo[1,2-b]pyridazine and pyrazolo[1,5-a]pyrimidine derivatives

#### Imidazo[1,2-b]pyridazines

**Figure 9.**
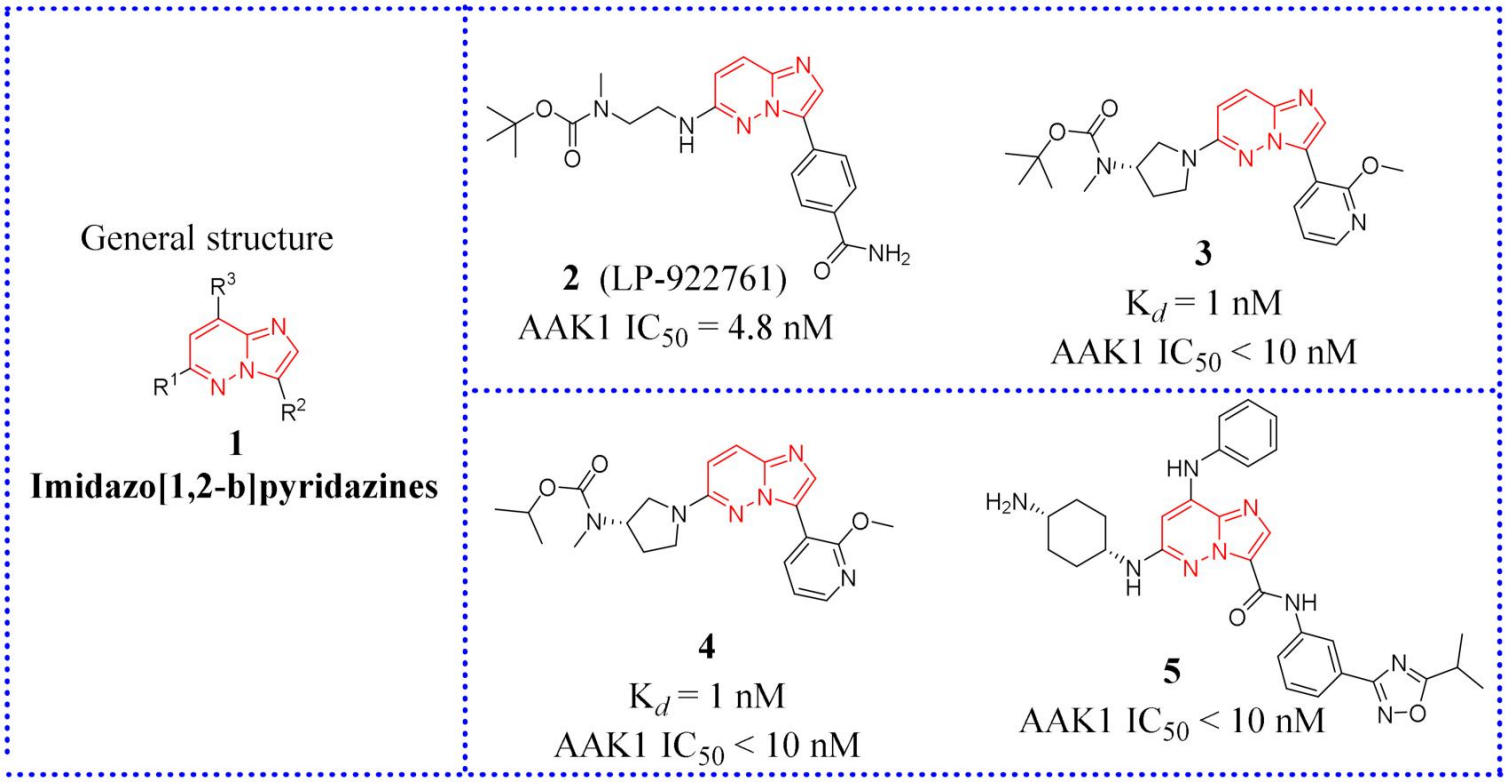
The chemical structures of patented AAK1 inhibitors with imidazo[1,2-*b*]pyridazine.

The general structure for the class of compounds depicted by **1** (Figure 9) contains a common fragment, immidazo[1,2-*b*]pyridazine. The compounds in this group have been reported in patent reviews, and this article summarises them only briefly.

This class of compounds was mainly pursued by Lexicon pharmaceuticals and Bristol Myers Squibb (BMS). Lexicon pharmaceuticals applied for two patents in 2013 (WO2013/134219, WO2013/134336), of which WO2013/134336 belongs to the patent utility category for medical use in the treatment of pain^59^^,^^60^. LP-922761 (compound **2**, Figure 9) is one of the effective means of relieving neuropathic pain. However, for compound **2**, oral administration still did not relief the pain. In this kind of structure, most of the active compounds carry a piperazinyl or pyrrolidinyl moiety at position 6, with the second nitrogen preferentially being derivatized as a carbamate moiety. At position 3, various aryl groups were introduced. Here a 2-methoxy-pyridinyl functionality often produces an active inhibitor (compound **3** and **4**, Figure 9). Unlike the derivatives disclosed by Lexicon pharmaceuticals, alkylamino, cycloalkylamino and phenylamino groups at position C8 of the core structure are claimed in the BMS patent (WO2015/026574)^61^. The patent declaration by BMS identifies seven imidazo[1,2-*b*]pyridazine compounds for which potent binding and inhibition of AAK1 were observed (IC_50_ < 10 nM). The disadvantage is that only the analogue without structural modification at position C8 (compound **5**, Figure 9) was tested and disclosed in this patent. At a dose of 60 mg/kg, this compound was less active than Gabapentin, when dosed at the equivalent 200 mg/kg (Gabapentin: The corresponding inhibition fraction at 200 mg/kg administered subcutaneously is 73%).

#### Pyrazolo[1,5-a]pyrimidine

**Figure 10.**
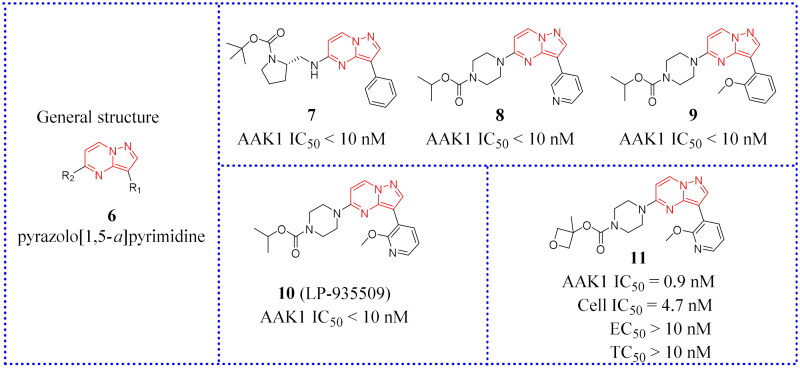
The chemical structures of patented AAK1 inhibitors with Pyrazolo[1,5-*a*]pyrimidine. EC_50_: median effective concentration, the concentration of drugs that causes 50% of the organisms to produce some toxic effect; TC_50_: median toxic dose, the drug dose that causes poisoning in half of the experimental animals.

Compared to Imidazo[1,2-b]pyridazines, this section focuses on compounds based on the imidazo[1,2-*b*]pyridazine scaffold in which the bridging nitrogen atom moves around the scaffold. The general structure is based on compound **6** (see Figure 10). Compounds in this group have been reported in patent reviews that this article only briefly summarises.

Lexicon pharmaceuticals proposed a series of pyrazolo[1,5-*a*]pyrimidine AAK1 inhibitors in the patent (WO2013/134228). Eighteen representative compounds that exhibited IC_50_ values of less than 10 nM were disclosed in the biochemical and cellular patent^62^. In this compound group, a piperazinyl or pyrrolidinyl functionality at position 6 and a (hetero)aryl at position 3 are the most common substituents. Among them, compound **7**, **8** and **9** (Figure 10) have the best inhibitory activity as demonstrated in a preclinical mouse model of neuropathic pain (the mouse formalin paw test). Compounds **7** and **8** are as active at a dose of 30 mg/kg (sc), as compared with Gabapentin at 200 mg/kg (sc). Compound **9** had good activity by oral administration (at 30 mg/kg sc), indicating good oral bioavailability, and **9** has also been reported in several reports in the biochemical literature.

Compound **10** (also known as LP-935509, Figure 10) is an effective, orally available, widely distributed and highly permeable AAK1 inhibitor^44^. Compound **10** inhibits the phosphorylation of μ2 protein (IC_50_ = 3.3 ± 0.7 nM, Cell IC_50_ = 2.8 ± 0.4 nM). In addition, **10** is competitive with ATP binding, having a K*_i_* value of 0.9 nM. It relieves neuropathic pain in various preclinical models of mice and rats, such as the CCI and streptozotocin (STZ) models. Unfortunately, it has no effect on acute pain. In short, compounds **10** is a promising candidates worthy of further study.

Lexicon pharmaceutical corporation synthesised compound **11** (Figure 10), and reported it to be a potentially important AAK1 inhibitor in the patent (WO2015/142714)^63^. The 3-methyloxetan-3-yl-4–(3-(2-methoxypyridin-3-yl)pyrazolo[1,5-*a*]pyrimidin-5-yl)piperazine-1-carboxylate showed potent activities with IC_50_ values of 0.9 nM in radio-active enzymatic and 4.7 nM in cell-based AAK1 assays. This compound demonstrated activity in both the formation assay and single nucleotide polymorphism SNP assay (also known as the Chung model). Bi et al. disclosed a number of analogues for AAK1 inhibitors based on pyrazolo[1,5-*a*]pyrimidine scaffolds with a carbon linker at position 5 in the patent (WO2015/035117)^64^. *In vivo* data are not discussed. Compound **11** was revealed to have certain antiviral utility in a separate patent application (WO2021/216454)^65^. The base compound was evaluated in three different *in vitro* cell models. Over the course of testing the efficacy in preventing infection by CoV-229E strain in MRC-5 cells, compound **11** exhibited good safety and low toxicity compared with the other three positive control drugs (EC_50_ > 10 μM; TC_50_ > 10 μM). In Huh‑7 cells, compound **11** and Sunitinib malate were used to test the anti-coronavirus activity of the OC43 strain of seasonal human oronavirus (HCoV). Results were characterised by a different anti-viral activity measure (by cytopathic effect, CPE) and cell viability was characterised by uptake of neutral red dye. The experimental results showed that compound **11** had similar antiviral outcomes to the positive control, but its cytotoxicity was much less. In addition, compound **11** appeared useful for reducing the viral load of HCoV-OC43 in H292 cells.

In addition, the effectiveness of a series of substituted compounds built upon the Pyrrolo[2,3-*b*]pyridine scaffold were summarised in a patent review^20^. The derivatives include: pyrrolo[2,1-*f*][1,2,4]triazine analogues, quinolines, and benzimidazoles. Then, the AAK1 inhibitors in the literature are discussed from the aspects of structure and rational design. Full considerations are given to the pharmacology and toxicology of the compounds of interest.

### Aryl amides

**Figure 11.**
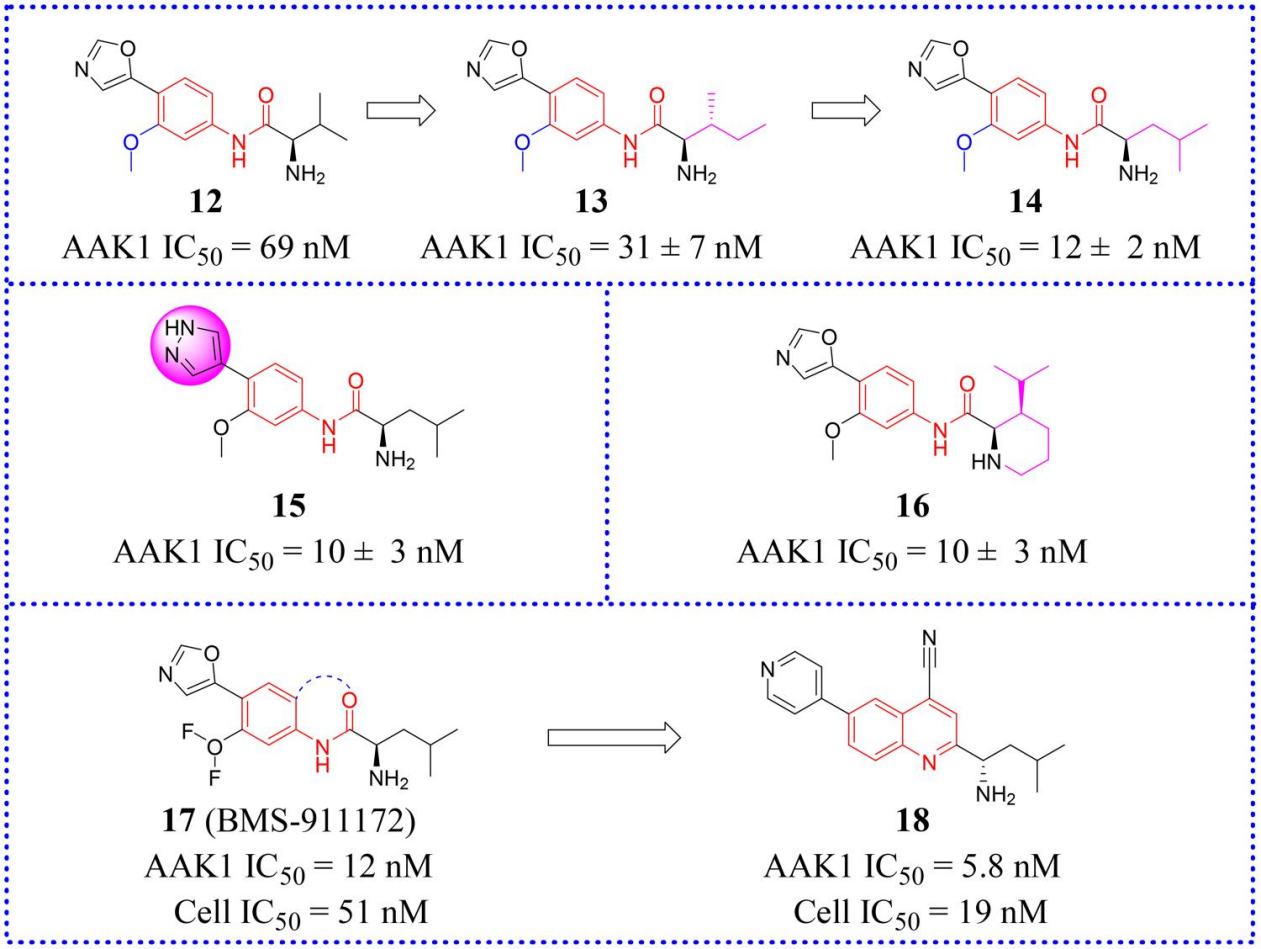
Structures of the AAK1 inhibitors with the aryl amide scaffold or its analogs selected from the references.

After screening their in-house compound library, Hartz et al. identified compound **12** (IC_50_ = 69 nM, Figure 11) as a potent lead compound with AAK1 inhibitory activity based on its desirable drug-like properties^66^. This compound had octanol: water partition coefficient cLogP = 0.93, topological polar surface area (tPSA) = 90 Å2, ligand efficiency (LE) = 0.47, and lipophilic ligand efficiency (LLE) = 6.2. Then, the structural modifications of compound **12** are focused on enhancing the initial inhibitory activity of AAK1.

**Figure 12.**
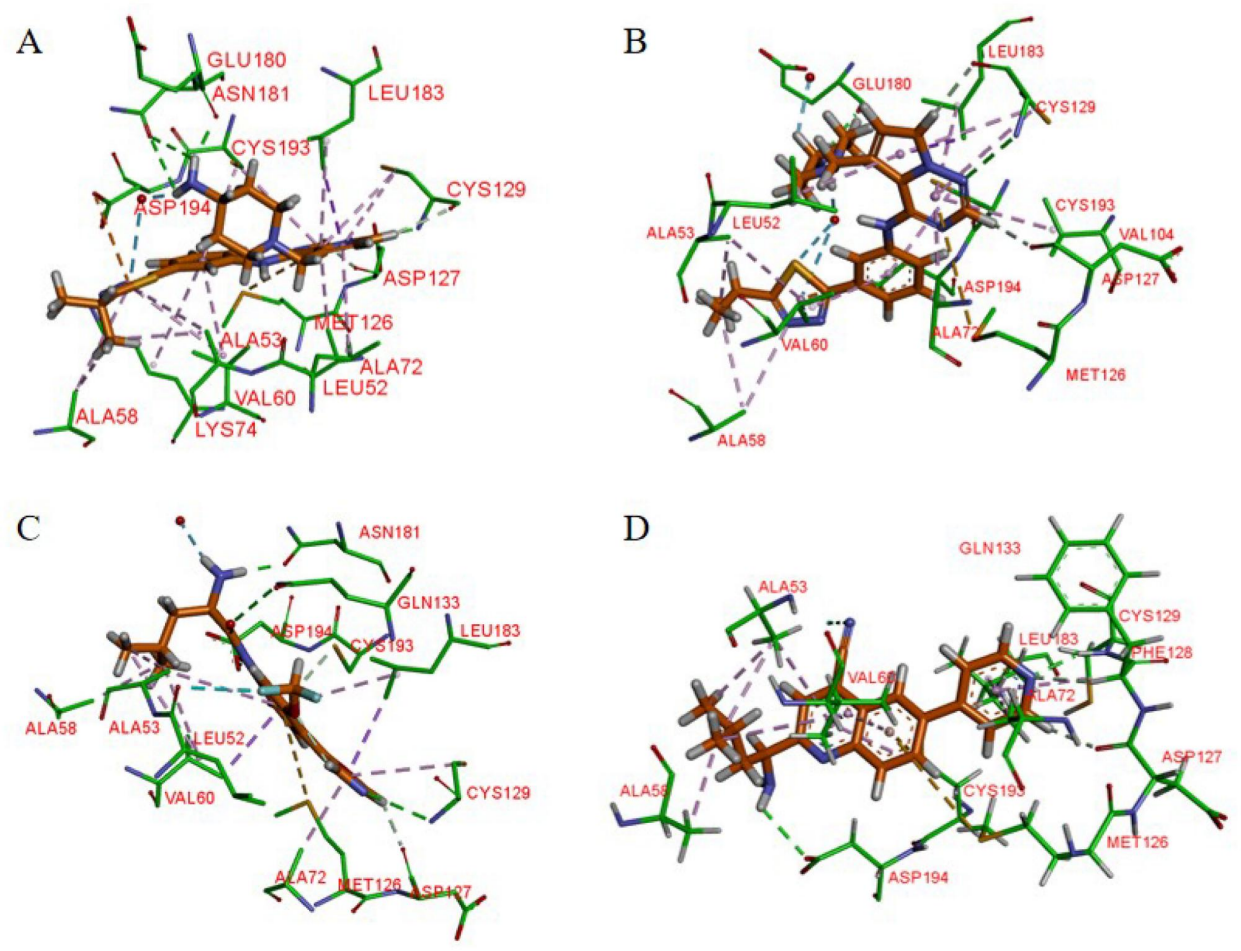
X-ray crystal structure of AAK1 in complex with compound **14** (PDB ID: 7LVH), **16** (PDB ID: 7LVI), **17** (PDB ID: 7RJ8) and **18** (PDB ID: 7RJ7). The compounds are rendered as a stick models with orange carbon atoms. Hydrogen bonds are shown as purple dashed lines.

Hartz et al. synthesised a series of AAK1 inhibitors based on **12** by attaching the different alkyl groups to the amide side capping group^66^. The introduction of a carbon chain with different lengths influences the activity of AAK1, and its activity will increase with the lengthening of the chain. By extending the alkyl side chain beyond the isopropyl group, the activity of AAK1 increased. Moreover, the addition of branches, such as analogues based on D-isoleucine, was found to lead to further improvements of inhibition. In addition, the substitution of the D-isoleucine-derived amide (**13**, IC_50_ = 31 ± 7 nM, Figure 11) by a D-leucine group (**14**, IC_50_ = 12 ± 2 nM, Figure 11) produced better efficacy. This substitution provided a lesson which may be drawn relating the size of side chains of the probe with respect to access to the ATP binding site of the kinase. Hartz et al. generated the X-ray crystal structure of AAK1 which showed the binding mode of compound **14** with 2.65 Å resolution^66^. From this structure, it can be found that the oxazole ring nitrogen forms a single hydrogen bond with the backbone NH of Cys129 within the kinase hinge region (see Figure 12). At the same time, the X-ray crystal structure of **14** also demonstrated that a key hydrophobic interaction occurs between the isopropyl group and a small lipophilic pocket in the P-loop region of the kinase, which could improve the potency and selectivity of the kinase. Further investigations revealed that replacing the methoxy group with a hydrogen results in a 13-fold reduction in potency. Hartz et al. attributed this to the loss of a hydrophobic contact between the Leu52 and Ala53 on the P-loop above and Leu183 below^66^. However, the side chain of D-leucine-capped amide seems to exhibit optimal hydrogen bonding interactions through the amide and amine nitrogens and an appropriate alkyl side chain length that promotes a stabilising bridging effect between the N-terminal domain (via the P-loop) and C-terminal domain (via the DFG motif) of the AAK1 kinase (Figure 12(A)). Based on the strength of the interaction between the hydrogen bond and kinase hinge region formed by oxazole nitrogen in **14**, they considered using similarly substituted heterocyclics to optimise such interactions. Of all the compounds tested, only pyrazole heterocyclic analog (i.e. **15**, IC_50_ = 15 ± 4 nM, Figure 11) had similar activity.

Subsequently, a structural constraint was introduced to the amide side chain of D-leucine derivatives, in which the flexible isobutyl group was tied back within a six-membered (piperidine) ring. It was also substituted with isopropyl at the 3-position. These modifications improved the potency and imparted a more rigid structure. Ultimately, compound **16** (IC_50_ = 10 ± 3 nM, Figure 11) was selected, which had a potency equivalent to **14**.

It is unfortunate that **16** had no follow-up progress to report due to its metabolic instability in mice. Eventually, a SAR analysis of substituents on the central benzene ring showed that **17** (IC_50_ = 12 nM, Figure 11) is more effective than compound **14**. Following a different line of investigation, they found compound **17** inhibited AAK1 activity by a more direct measurement of the phosphorylation of μ2. Using a Western blot analysis of the μ2 subunit and showed 82% reduction of μ2 phosphorylation following acute administration of compound **17**, *vs* 90% for control. The EC_50_ of inhibition of μ2 phosphorylation in vivo was determined to be 8.3 μM, resulting in an ED_50_ of 6 mg/kg. Kinase selectivity profiling of compound **17** against a panel of 219 kinases at 1 μM showed the compound had good overall kinase selectivity. By comparing their pharmacokinetic (PK) parameters their compound’s (**14**, **15**, **17**) pharmacokinetic parameters in male G57BL6 mice, compound **17** was identified as the excellent inhibitor.

Due to its ability to form hydrogen bonds within the ATP binding site at the hinge region (see Figure 12(C)), compound **17** becomes a potentially ATP-competitive small molecule kinase inhibitor. Compound **17** (later named BMS-911172) demonstrated the following favourable pharmacologic properties: a clogP = 1.9, tPSA = 90 Å^2^, LE = 0.45 and LLE = 6.0. The aqueous solubility of **17** at pH 1 and pH 7.4 was 5.85 mg/mL and 0.60 mg/mL, respectively. An increasing volume of *in vivo* measurements found that **17** significantly reduces phase II hind paw flinching and licking/biting in the formalin pain model. In the CCI model, compound **17** (60 mg/kg, sc) significantly reduced hyperalgesia, with the maximal reversal increased by 24% (thermal) and 13% (mechanical) with respect to the positive comparator gabapentin.

Subsequently, new work was reported in 2022 in which novel compounds possessing quinazoline and quinoline structures that differed from compound **17**. Hartz continued to modify **17** to obtain compounds with better activity. The new compounds transferred the amide of **17** back to the neighbouring phenyl ring to form a bicyclic benzene-pyridine structures, then modified the synthesis to produce a series of fused pyridine-benzene (quinoline) heterocycles and bicyclic pyridine compounds. Among them, a selective potent AAK1 kinase inhibitor **18** (Figure 11) was discovered, which improved enzyme and cellular potency compared to **14**. *In vitro* testing in a HEK cell culture showed that **18** is the most potent compound with an IC_50_ value of 19 nM. Compound **18** also presented favourable metabolic stability^67^. Compound **18** had high permeability across membranes, which allowed it to penetrate into the central nervous system (CNS). Its LE value is 0.47 and LLE value is 5.4. Compound **18** also showed good overall selectivity in a kinome panel screening (using 403 kinases). Because of its potent capacity to inhibit the phosphorylation of μ2 in mouse brain, it was also regarded as a likely candidate for safe testing in human subjects because drug exposures can be kept low and still be effective. Therefore, **18** seemed to be a candidate potential for clinical development or further preclinical research.

### Bis(hetero)aryl ethers

**Figure 13.**
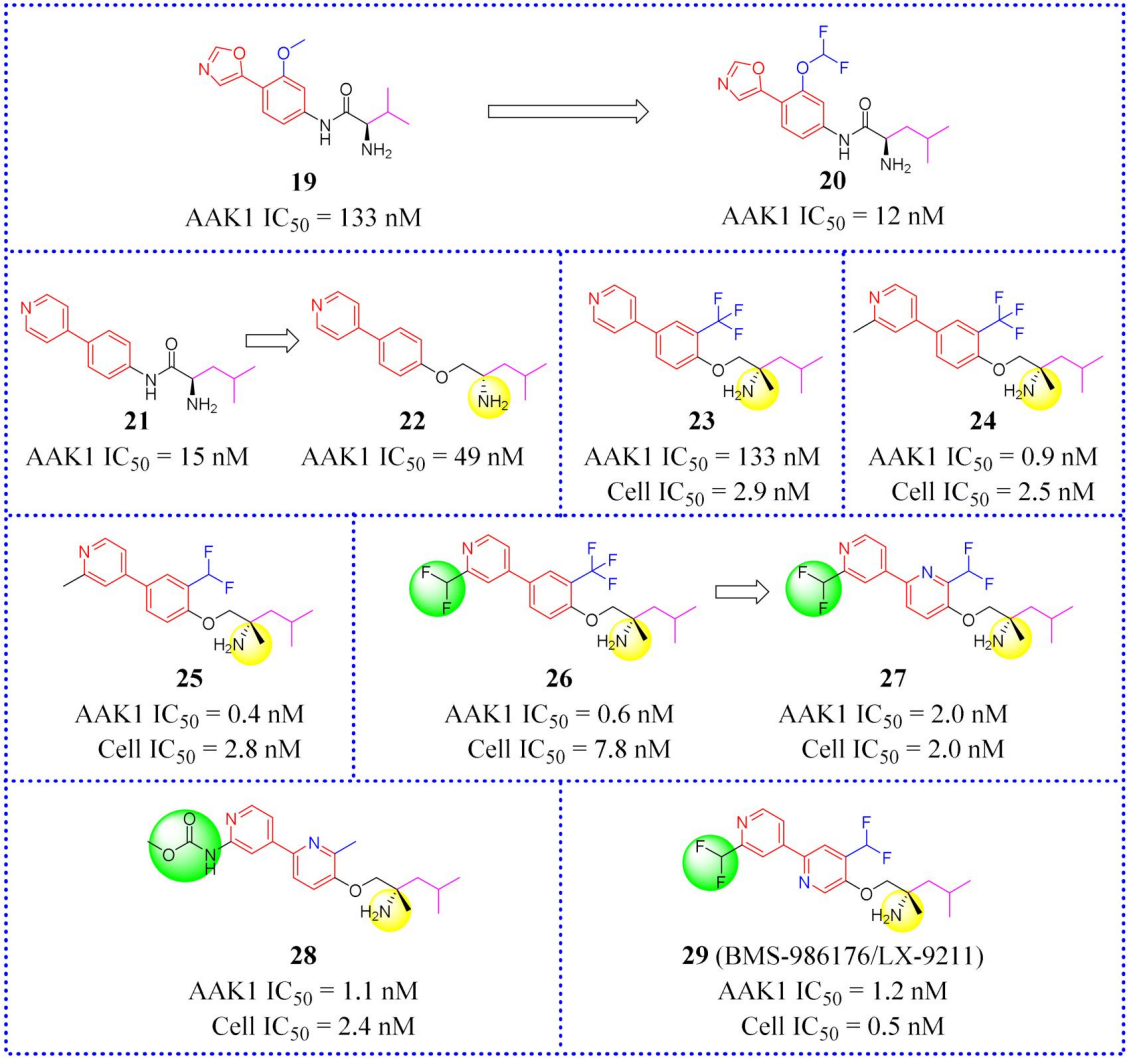
Structures of the AAK1 inhibitors with the Bis(hetero)aryl ethers or their analogs selected from Ref. 11, 68.

Luo et al. first reported a series compounds containing a bis(hetero)aryl scaffold as inhibitors of AAK1^11^. These compounds against AAK1 were tested and their activities were mostly in the Nano molar range. In addition, the oxazole-phenyl compound **19** was screened (Figure 13) as the lead against AAK1 using a high-throughput screening campaign. And it could be found that compound (**20**, Figure 13) to be optimal after conducting an extensive SAR study of compounds bearing biaryl and amino-amide chains^11^. In the CCI model, **20** reduced the nociceptive response induced by phase II formalin in rats. This is a reaction known to be closely associated with the phosphorylation level of μ2. In addition, chemical studies confirmed that the key hydrogen-bonding interaction occurred between the oxazole head group of compound **20** and the AAK1 hinge binding site. When other heterocyclic substituted oxazoles were found to be effective inhibitors, only pyridyl (**21**, IC_50_ = 15 nM, Figure 13) was well tolerated and its efficacy was equivalent to **20**. When the amide group was substituted by other groups (such as an ether group), the potency decreases, presumably due to the destruction of the hydrogen-bond-linkage between the amide NH and AAK1. However, the potency of **22** (IC_50_ = 49 nM, Figure 13) that carried additional fluorocarbon groups was still within the acceptable range. Lu made a full SAR study on the ether derivative **22**. Encouragingly, compound **22** is more effective, and its IC_50_ is 49 nM. For this reason, compound **22** appeared to be a promising lead compound.

CYP3A4 (Cytochrome P4503A4 enzyme) is one of the members of the cytochrome P450 oxidase family that is the major enzyme for the detoxification of bile acids. Eliminating CYP3A4 inhibition is a problem that is often considered in drug research and development. It mainly exists in the small intestine and liver, and can oxidise foreign organic small molecules, such as toxins or drugs, so that they can be excreted. CYP3A4 enzyme inactivates many drugs, so before testing the drugs effect in vivo it is frequently necessary to inhibit CYP3A4. Compounds that show excellent potency for inhibiting AAK1 (**23**, IC_50_ = 0.6 nM and Cell IC_50_ = 2.9 nM, Figure 13) may also have CYP3A4 inhibition problems. In the course of *in vivo* testing, Luo and his group discovered that compound **23** was susceptible to CYP3A4 inactivation.

Subsequently, Luo found that adding a methyl group beside the chiral amine (in **21**, Figure 13), or placing it next to a pyridyl nitrogen (**24**, **25**, Figure 13) avoids CYP3A4 inhibition. This is a major discovery. In DPNP model testing, compound **26** (Figure 13) was shown to be worthy of attention because of its good permeability across the CNS and the high drug in the brain vs drug in plasma ratio (b/p ratio = 11). Unfortunately, **26** may be susceptible to some problems such as oral exposure and CV liabilities. Moreover, the lack of a good safety profile prevented it from entering clinical trials. This prompted medicinal chemists to further optimise the core in order to identify more active allosteric inhibitors. To solve the potential problem of CV liabilities, 2-pyridyl, and 3-pyridyl analogues were synthesised and a series of inhibitors were obtained. Among these, representative compound **27** (Figure 13) not only possesses good cell potency (IC_50_ = 2.0 nM) but also shows good metabolic stability and AR relaxation of 42%. Compound **27** showed good kinase selectivity in our internal 249 kinase panels. In the CCI model, the pain response of **27** was reversed by 97% at the dose of 1 mg/kg. However, the lowest effective dose is 0.3 mg/kg. At effective dose levels, compound **27** was less likely to produce sports injuries than gabapentin.

A potent and selective radio ligand of the [^3^H]BMT-046091 is applied to determine AAK1 distribution and target engagement. An *in vitro* [^3^H]BMT-046091 binding autoradiography assay determined the spinal cord occupancy rate of compound **27** to enable a better understanding of the relationship between AAK1 target engagement and the anti-neuropathic pain effects. The results showed good target binding in the spinal cord. In the diabetic peripheral neuropathic pain (DPNP) rat model, oral administration of **27** significantly reduces mechanical abnormal pain, and the inhibition rate of pain response is more than 60% at the dose of 1 mg/kg. Compound **27** (i.e. BMS-986176, renamed as LX-9211 by Lexicon) had entered into clinical phase II research studies and is currently aimed at delivering palliative relief to patients with diabetic peripheral neuropathic pain and patients with postherpetic neuralgia^11^.

Subsequently, **28** and **29** (Figure 13) were tested head-to-head with **27**. Both of them showed higher potency against AAK1 and were effective in CCI and DPNP rat pain models. Unfortunately, a degree of hepatotoxicity and other CV liabilities at high doses prevented these two compounds from further development^68^. Although **28** and **29** still have some problems, the results of this study provide a new scaffold for further developing novel AAK1 inhibitors.

## AAK1 inhibitor for inhibiting virus

AAK1 is a potential antiviral biological target. Up to now, no AAK1 inhibitor has been approved for antiviral indications. In this section, the structure and antiviral activity of AAK1 inhibitors with antiviral effect are systematically summarised, which provides a reference for the subsequent research on developing antiviral drugs targeting AAK1.

### Repurposing of other kinase inhibitors

#### Sunitinib

**Figure 14.**
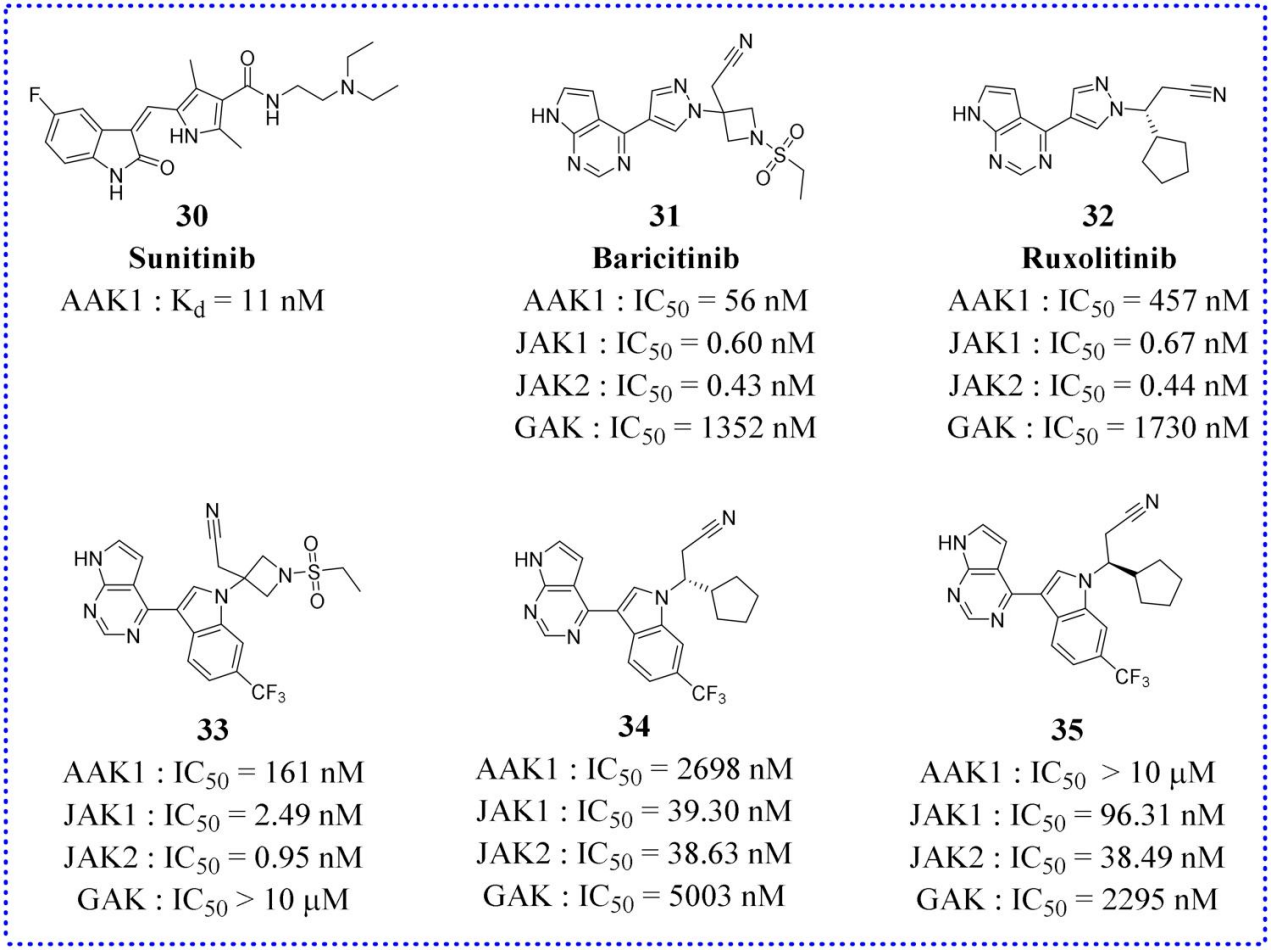
Chemical structures of approved drugs **30**–**32** and their derivatives **33**–**35**.

Sunitinib (**30**, Figure 14) was approved by the FDA in 2006 for the treatment of gastrointestinal stromal tumours (GIST) and renal cell carcinoma (RCC). However, **30** maintains a nanomolar affinity for AAK1 and had been widely used to study the role of AAK1 in virus infection (see Table 1). Einav’s research group found that **30** showed broad-spectrum anti-viral activity against a variety of viruses^51^. The EC_50_ values for most viruses were below 1 μM, and the CC_50_ values were all greater than 10 μM. (The CC_50_ (for cytotoxic concentration) value indicates the toxicity to normal cells). The higher of value means better safety.

**Table 1.** Anti-viral activity of **30**^51^.

Virus	EC_50_ (μM)	CC_50_ (μM)	Cells
HCV	1.20	>10.00	Huh 7.5
DENV1	0.60	>10.00	BHK-21
DENV2	0.51	11.50	Huh 7.5, BHK-21
DENV3	0.30	>10.00	BHK-21
DENV4	0.23	>10.00	BHK-21
WNV	0.55	>20.00	MEF, Vero
ZIKV	0.51	14.10	Huh 7.5
EBOV	0.47	>10.00	Huh 7.5, Vero
JUNV	4.80	10.40	Vero
HIV	0.80	>20.00	HeLa, TZM-b1
RSV	<0.12	12.50	Hep2
CHIKV	4.67	11.90	Vero

EC_50_: Half-maximal effective concentration; CC_50_: Half-maximal cellular cytotoxicity; HCV: Hepatitis C virus; DENV: Dengue virus; WNV: West Nile virus; ZIKV: Zika virus; EBOV: Ebolavirus; JUNV: Junin virus; HIV: Human immunodeficiency virus; RSV: Respiratory syncytial virus; CHIKV: Chikungunya virus.

#### Baricitinib

Baricitinib (**31**, Figure 14) was approved by the FDA for the treatment of rheumatoid arthritis in 2018. As early as 2016, Sorrell et al. found that **31** had a strong inhibitory effect on AAK1^3^. The results of isothermal titration calorimetry (ITC) showed that the K*_d_* value of **31** on AAK1 reached 17.2 nM. Recently, the Stebbing team analysed and calculated the inhibitor drugs on the market using BenevolentAI’s unique database and artificial intelligence algorithm^52^. In the literature, it is recommended that **31** be tested to block the process of virus infection, because of an anticipated property to interfere with endocytic viral entry in lung cells. Later, the *in vitro* pharmacology and *in vivo* pharmacokinetics of **31** in relevant white blood cell subset were evaluated. It was found to inhibit the signal transduction of cytokines associated with the novel coronavirus infection. Compound **31**, predicted by AI, weas verified to have a positive biochemical inhibition on human NAKs family members, and its K*_d_* values on AAK1 was determined to be 8.2 nM at the same time. There are currently twenty-six clinical studies underway to examine the role of **31** for the treatment of novel coronaviruses, utilising searches of the FDA clinical database. According to the FDA’s official website, five of them have completed phase II/III clinical trials (NCT04421027, NCT04358614, NCT04362943, NCT04401579, and NCT04640168). The combination of **31** and the anti-viral drug Remdesivir may be an appropriate combination therapy strategy. Investigators have been concerned about the risk of adverse effects such as immunosuppression, secondary infection, and thrombosis using this combination regimen. Thankfully, clinical studies have shown fewer acceptable adverse effects.

#### Analogs derived from clinical compound

In addition to the above-mentioned kinase inhibitors that have been marketed for clinical use, there are also some newly synthesised small molecule inhibitors that are being investigated in preclinical studies. For example, on the basis of Ruxolitinib (**32**, Figure 14) and **31**, Lin et al. designed some small molecule analogues using a scaffold jumping strategy (compounds **33**, **34,** and **35** in Figure 14)^58^. Replacing the pyrazole ring in **31** with a trifluoromethyl substituted indole ring gave compound **33** with comparable potency for AAK1 (IC_50_ = 161 nM), JAK1 (IC_50_ = 2.49 nM), and JAK2 (IC_50_ = 0.95 nM). However, **33** showes much weaker inhibition against GAK (IC_50_ > 10 μM) than **31** displayed (IC_50_ = 1.35 μM), which indicates that the substitution of the pyrazole ring by a trifluoromethyl substituted indole ring reduces the modified inhibitor’s affinity towards GAK. The reason may be the introduction of a fused trifluoromethyl phenyl group produces a steric hindrance with Lys69 and Cys190 of GAK. Compared with **31**, the selectivity of **33** for AAK1, JAK1, and JAK2 is better than for GAK. However, its selectivity may not be compatible with the anti-coronavirus effect. On the other hand, the improvement in the selectivity of **33**, particularly against AAK and JAK, may be more useful for intervention in other diseases, such as autoimmune diseases, where GAK inhibition is not deemed to be useful. Increased selectivity reduces the risk of toxicity caused by less off-target binding. Sadly, compound **34** showed slightly decreased (3–6-fold) potency for binding to AAK1 and GAK but dramatically decreased (59–88-fold) potency for JAK1/2 compared to **32**. Similar to **34**, compound **35** was 87–144-fold less effective than **32** for binding to JAK1/2. Compared to **32**, **35** showed comparable binding to GAK, but a 22-fold lower affinity for AAK1. Ultimately, compound **33** warranted further study in more advanced preclinical studies to fully understand its potential as an effective anti-viral therapy.

### Pyrrolo[2,3-b]pyridines

**Figure 15.**
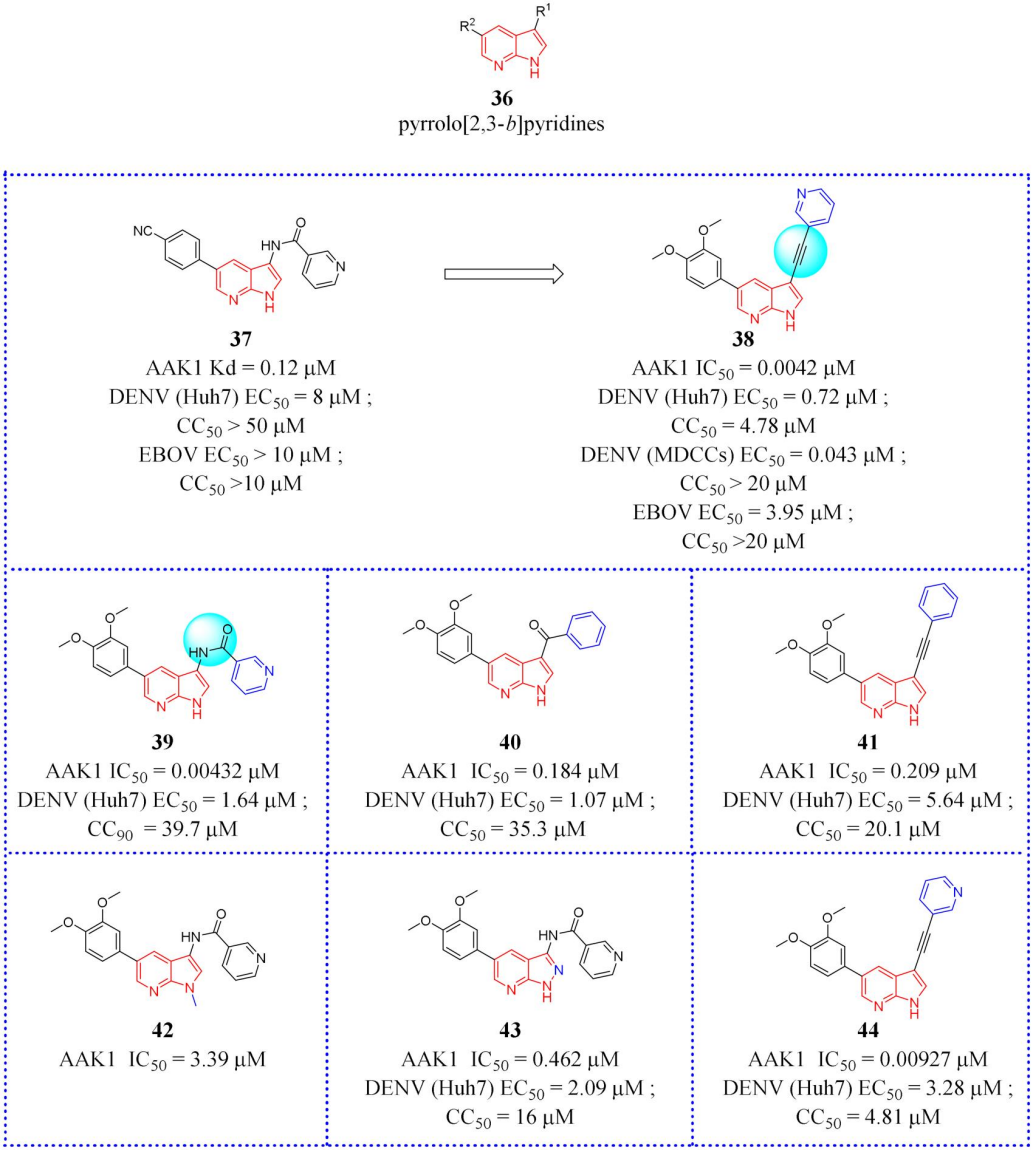
Structures of the AAK1 inhibitors based on the pyrrolo[2,3-*b*]pyridine scaffold or its analogs as selected from the references.

This section focuses on AAK1 inhibitors bearing a pyrrolo[2,3-*b*]pyridine scaffold^69^. The general structure of pyrrolo[2,3-*b*]pyridines-based analogues is shown in compound **36** (Figure 15).

**Figure 16.**
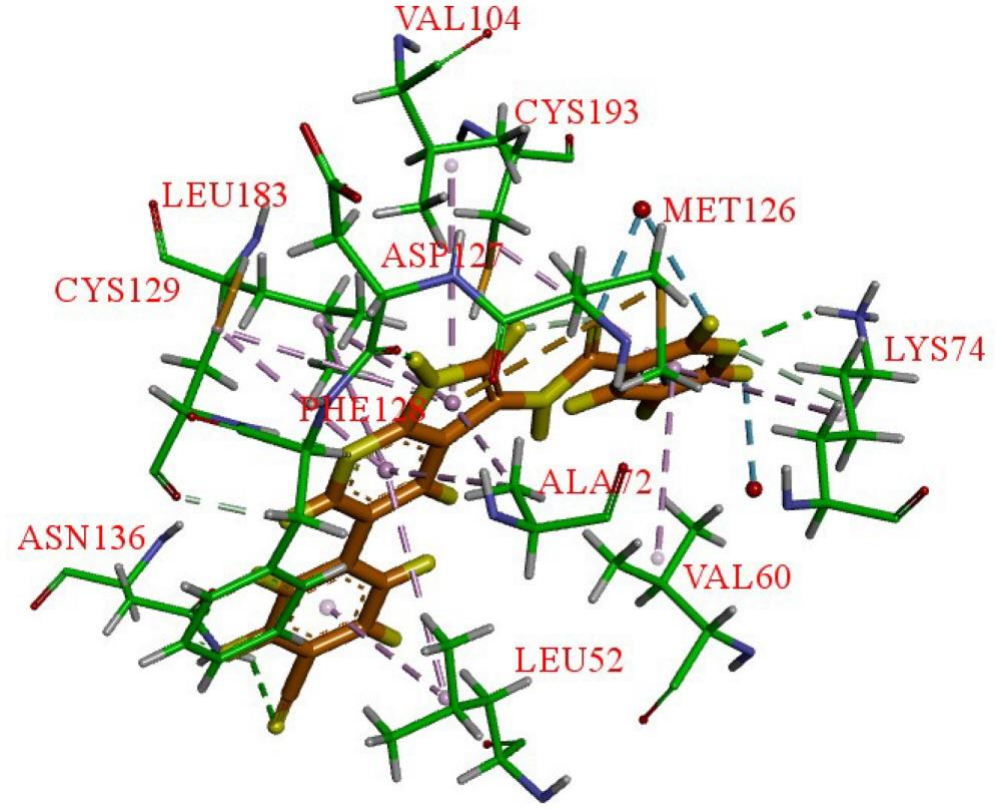
X-ray crystal structure of AAK1 in complex with compound **37** (PDB ID: 5L4Q). The compound is rendered as a stick model with orange carbon atoms. Hydrogen bonds are shown as purple dashed lines.

Using the DiscoverX binding assay format, Verdonck et al. tested an unsubstituted pyrrolo[2,3-*b*]pyridine scaffold and a 7-aza-indole derivative (**37**, Figure 15) as novel AAK1i candidates with nanomolar potency (K*_d_* = 53 nM)^69^. And a series of 577 structurally diverse compounds were screened across a panel of 203 protein kinases. The results of binding-displacement assays show that compound **37** was 3-fold more selective for AAK1 over GAK, 8-fold more selective for AAK1 over BMP2K, and 22-fold more selective for AAK1 over STK16. In the crystal structure of compound **37** bound to AAK1 to 20 Å resolution, the nitrogen atoms of the pyrrolo[2,3-*b*]pyridine moiety form two hydrogen bonds to the peptide backbone of residues Asp127 and Cys129 that are located at the kinase hinge region. The 4-cyanophenyl moiety is oriented towards the solvent with the phenyl ring directly sandwiched between the backbone of Gly132 of the hinge and Leu52 of β1 in the N-lobe. The nitrogen of the cyano moiety forms a polar interaction with the side-chain of Asn136 (Figure 16). Therefore, it is a promising lead that can be further optimised.

When the 4-cyanophenyl derivative in **37** was replaced with a 3,4-dimethoxy-phenyl functionality, compound **39** (Figure 15) was resulted. **39** showed stronger binding affinity for AAK1 and also demonstrated the most effective anti-DENV activity (EC_50_ = 1.64 μM, EC_90_ = 7.46 μM). Verdonck et al. carefully studied the structure-activity relationship (SAR) of the above derivatives^69^. Then, the inhibition effect was examined after the replacement of the 3-pyridyl group by a number of (hetero) aromatics and cycloaliphatic groups. These compounds demonstrated a 100-fold reduced affinity for AAK1 IC_50_ values ranged between 0.1–0.6 μM and antiviral activity relative to compound **39** was diminished, indicating the important role of 3-pyridyl moiety for biological activity. Furthermore, comparative inhibition studies showed that the amide moiety is not necessary for AAK1 binding and can be substituted with other groups, such as ketone (**40**, Figure 15) or alkyne (**41**, Figure 15). Verdonck et al. synthesised an innovative series of substituted phenylacetylene derivatives and found that the introduction of a 3-pyridylacetylene (**38**, Figure 15) significantly improves the affinity of AAK1 (IC_50_ = 0.0042 μM), enhances anti-viral activity (EC_50_ = 0.72 μM), and exhibits only a moderate cytotoxic effect (CC_50_ = 17 μM)^65^. Therefore, **38** appeared to be a candidate potential for clinical development or for further pre-clinical investigation.

In subsequent work, Verdonck et al. synthesised three different compounds based on the 7-aza-indole scaffold (compound **37**)^69^. Methylation of the pyrrole nitrogen on compound **39** produced compound **42** (Figure 15). In addition, a pyrazolo[3,4-*b*]pyridine analogue (**43**, Figure 15), and a pyrrolo[2,3-*b*]pyrazine analogue (**40**, Figure 15) were also synthesised. Among these, compounds **42** and **43** showed low AAK1 affinity (> 800-fold and 100-fold loss in activity compared to compound **39**). The significantly enhanced cytotoxicity (by 3-fold) of compound **44** was showed in Huh7 cells. Compounds **37**, **38** and **39** were tested for their activity against the unrelated EBOV to evaluate the potential for broad-spectrum antiviral coverage. The biological assay showed that compound **38** possessed low nanomolar potency and excellent anti-EBOV (Ebola) activity with EC_50_ and EC_90_ values of 0.0428 μM and 1.49 μM, which its mechanism was to regulate the phosphorylation of AP2M1 via AAK1 inhibition. These assays demonstrated that **38** was an effective inhibitor regulating viral uptake in the treatment of a broad spectrum of viral agents. Unfortunately, the KINOMEScan assay (Discover X) showed that **38** was non-selective as an AAK1 inhibitor. In addition to AAK1, compound **38** targets multiple other kinases, which might contribute to its antiviral effects. Therefore, in the future, pharmacists can use compound **38** as a lead compound to optimise its structure and improve its selectivity.

## Other AAK inhibitors

### 1H-indazole analogues

**Figure 17.**
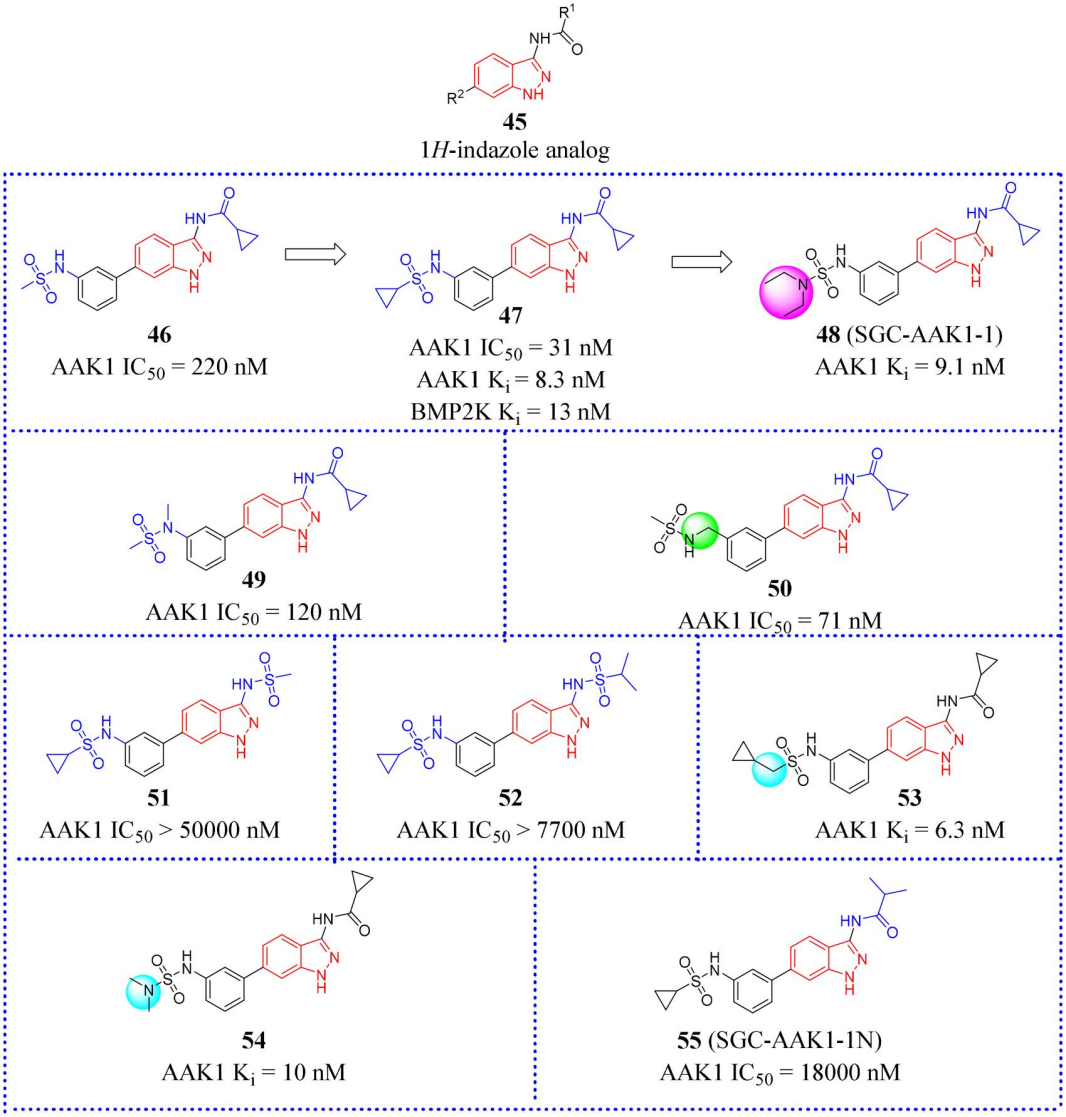
Structures of the AAK1 inhibitors with the 1*H*-indazole scaffold or its analogs selected from the references.

The base scaffold structure of compounds in the class is shown as **45** (Figure 17). Well et al. developed a series of novel 3-acylaminoindazole derivatives as a small molecule chemical probe (SGC-AAK1-1, **48**, Figure 17). Compound **45** bound with strong affinity for AAK1/BMP2K over other AAK1 family members^70^. Compound **48** was discovered to possess high affinity for both AAK1 and BMP2K with K*_i_* values of 9.1 and 17 nM, respectively. The effect of compound **48** on WNT signalling was recently reported. Novel 3-acylaminoindazole analogues were designed and synthesised. These afforded a series of modified compounds that could be assayed for the strength of binding and their ability to inhibit phosphorylation by WNT and other family members. Scientists hope to improve the cell activity by changing the substituents on sulphonamides. On the other hand, different lengths of alkyl chains or alkylamines were used as the linker to explore the conformation effects of the target molecules. From these data, it was determined that a sulphonamide moiety appending at the 3-position of the aryl ring produced the greatest AAK1 inhibition. In addition, sulphonamides with small saturated rings (< 6 atoms) that was mono-substituted by short alkyl chains or alkylamines also increased the activity against AAK1^70^. It is preferable to make modifications at the C3 position of the aryl ring than at the C4 position of the indazole scaffold. This allows a hydrogen-bonding network to become established in the ATP-binding pocket, which can be entered by 3-position groups. Therefore, the C3 position of the aryl ring is the key site for establishing a hydrogen bond with the ATP binding pocket. Then these become capable of donating a hydrogen bond and provide favourable interactions with the DFG motif aspartate residue (Asp198/194). Small acidic groups at these positions are not favoured due to charge repulsion^70^. So the interaction between charges has an important impact on the structure of matter and molecular changes.

*N*-Methylation is a common method in the process of chemical compound design. Not surprisingly, methylation of the aryl 3-nitrogen (**49**, IC_50_ = 120 nM, Figure 17) or insertion of a methylene spacer between the sulphonamide and aryl ring (**50**, IC_50_ = 71 nM, Figure 17) increased potency for AAK1. By contrast, the substituent at the indazole 3-position appeared to form a polar interaction with the kinase hinge region that was sensitive to structural modifications. Sulphonamides with cyclopropyl substituents (**47**, IC_50_ = 31 nM, Figure 17) were selected for further SAR studies. Due to steric hindrance and charge interactions caused by the proximity of the sulphonamide oxygen atoms with nearby protein side chains, it was not advisable to replace amide with sulphonamide groups (eg. **51** and **52**, Figure 17)^70^. Co-crystal structures reveal that the group attached to the 3-position amide exposes solvent at this position, which could explain the moderate tolerance of AAK1 for larger and bulkier substituents.

To explore the effect of alkyl amine or longer chain hydrocarbon substituents on AAK1 activity, two additional 3-pacylaminoinda-zoles (**53** and **54**, Figure 13)^70^ were designed. Similar to compound **47**, compounds **53** and **54** did show high inhibition for AAK1 at 1 μM in the split luciferase assay, inhibiting AAK1 by 93% and 94% for **54** and **55**, respectively. Moreover, the TR-FRET binding-displacement assays showed that they are dual AAK1/BMP2K inhibitors with greater than 5-fold selectivity over STK16 and 50-fold selectivity over GAK. The only successful co-crystal structures were obtained with BMP2K-KD, while co-crystals of acylaminoindazoles bound to the AAK1-KD were not successful^70^. To enhance the utility of dual AAK1/BMP2K probes, Well et al. designed a series of effective inhibitors by incorporating other caps such as fluorinated aryl rings, alkyl chain capped with fluorine groups, small hydrocarbon chains and rings, and alkyl amines linked to the sulphonamide^70^. Fifteen compounds were synthesised using different synthesis strategies to assemble the heterocycle caps. Among these, compound **48** (Figure 17) was purified and showed a high affinity of this series for AAK1 and BMP2K. **48** demonstrated at least 35-fold selectivity for BMP2K over GAK and 4–16-fold selectivity for BMP2K over STK16. The KINOMEscan (DiscoverX) assay against 403 wild type human protein kinases at 1 μM showed that compound **48** exhibited good binding affinity (K*_d_*) of all kinases inhibited >80%. In addition, compound **48** was designated as a small molecule inhibitor probe that targets the ATP-binding site of AAK1 and BMP2K preferentially over other serine/threonine kinases. The complementary negative control analog compound **55** for this probe (also called SGC-AAK1-1N, Figure 17) was made commercially available, becoming the only chemical probe targeting AAK1 that is widely used in conjunction with its negative control. In short, the results provide a new scaffold for the further research of developing novel probe.

## Conclusions and perspectives

As a specific key kinase of the NAKs family that regulates the phosphorylation of threonine at the 156th position of the μ2 subunit of AP-2 protein, AAK1 plays a crucial role in CME. And the phosphorylation of Thr156 plays an important role in the regulation of protein structure and function, such as affecting downstream signals, apoptosis, and differentiation. Further study of AAK1 plays an important role in exploring the physiological and pathological mechanisms in cells and promote the treatment and prevention of related diseases. Because AAK1 is expressed in both the membrane and cytoplasm of normal cells, its internalised substances come from the external environment, such as micronutrients, and even some viruses and bacteria. Therefore, there are some potential safety problems when developing small molecular inhibitors. However, AAK1i has already been proved to have certain activity in clinic, suggesting it will not cause irreversible harm to the human body. Nevertheless, since it is not clear whether interference of this endocytosis pathway to mitigate pathological and physiological conditions will eventually lead to adverse side effects, small molecule compounds should be designed with safety in mind. These objections may be an explanation for the limited drug discovery activity against AAK1. There are two main areas of interest in the development of AAK1 inhibitors, one for pain therapy and the other for viral infections. At present, only Lexicon and BMS pharmaceuticals are active in these two fields. Since few companies are active in AAK1 drug discovery, the limited structural diversity of AAK1 inhibitors has been explored. In the field of pain treatment, great progress has been made, and the existing compounds have reached Phase II clinical studies. On the virology front, the COVID-19 pandemic has significantly increased the scientific interests in AAK1 protein for treating virus infection. Consequently, the interest in AAK1 inhibitors development, as reflected in a number of publications, has seen a major hike in recent years. Despite the important role of the AAK1 protein in the clathrin-mediated endocytosis of virus invasion, more work is needed including studies on aspects of kinase selectivity, safety window, and anti-virus effectiveness. To date, no AAK1 inhibitor has been approved for therapeutic uses including for the treatment of emerging viral infections, such as Dengue, Ebola and novel coronaviruses.

Another issue that needs to be addressed is kinase selectivity. Among the existing various kinases, their ATP pockets are similar. It may lead to the development of small molecule compounds that act on other kinases and even cause off-target effects. Therefore, from the stand point of medicinal chemistry and drug discovery, the development of AAK1 inhibitors based on rational drug design is practical and productive. This is because the crystal structure of the AAK1 protein has been solved. Therefore, the structural characteristics and structure/activity relationships appear to be straightforward and easy to understand. In this paper, the up-to-date advances of AAK1i in medicinal chemistry are summarised with the hope of providing the scientific community with a quick knowledge reference to accelerate their drug discovery. A number of studies reported that AAK1i have shown efficacy in various preclinical animal models. Among all the animal models, the neuropathic pain model has been studied most extensively. On the other hand, more in-depth studies are required to understand the mechanism and the role of AAK1i in preventing or inhibiting virus entry into host cells. The greater breakthrough efforts in this area are required to facilitate identification of safe and effective mediators for potential anti-virus therapy against emerging infections, which is highly anticipated.

## CRediT authorship contribution statement

**Ying-Yui Yuan:** Literature searching, Methodology, Resources, Data curation, Writing - original draft, Visualisation. **Nian-Dong, Mao:** Methodology, Resources, Data curation, Writing - original draft. **Ji-Long Duan:** Resources, Data curation. **Hang Zhang:** Graphs preparation. **Carmen Garrido:** Editing, Visualisation. **Frédéric Lirussi:** Editing, Visualisation. **Yuan Gao:** Supervision, Writing - review & editing, Visualisation. **Tian Xie:** Methodology, Conceptualisation. **Xiang-Yang Ye:** Conceptualisation, Writing - review & editing, Project administration.
